# Distribution and dynamics of Greenland subglacial lakes

**DOI:** 10.1038/s41467-019-10821-w

**Published:** 2019-06-26

**Authors:** J. S. Bowling, S. J. Livingstone, A. J. Sole, W. Chu

**Affiliations:** 1 Lancaster Environment Centre, Lancaster University, Lancaster, LA1 4YQ UK; 2 Department of Geography, University of Sheffield, Sheffield, S10 2TN UK; 3 Department of Geophysics, Stanford University, Stanford, CA 94305 USA

**Keywords:** Environmental sciences, Hydrology

## Abstract

Few subglacial lakes have been identified beneath the Greenland Ice Sheet (GrIS) despite extensive documentation in Antarctica, where periodic release of water can impact ice flow. Here we present an ice-sheet-wide survey of Greenland subglacial lakes, identifying 54 candidates from airborne radio-echo sounding, and 2 lakes from ice-surface elevation changes. These range from 0.2–5.9 km in length, and are mostly distributed away from ice divides, beneath relatively slow-moving ice. Based on our results and previous observations, we suggest three zones of formation: stable lakes in northern and eastern regions above the Equilibrium Line Altitude (ELA) but away from the interior; hydrologically-active lakes near the ELA recharged by surface meltwater and; small, seasonally-active lakes below the ELA, which form over winter and drain during the melt season. These observations provide important constraints on the GrIS's basal thermal regime and help refine our understanding of the subglacial hydrological system.

## Abstract (web summary)

Subglacial lakes can influence basal hydrology and ice flow in Antarctica, but are poorly constrained in Greenland. Here the authors provide the first ice sheet-wide inventory of subglacial lakes beneath GrIS, including 54 uncharted lakes.

## Introduction

Only four subglacial lakes have been discovered beneath the Greenland Ice Sheet (GrIS), despite evidence suggesting a significant proportion of the bed is thawed^1,2^ and that some of this basal water forms metre-scale diameter ponds over ~3 cm deep^3^. Two small (<10 km^2^) lakes, separated by a bedrock island, were detected beneath the Bowdoin Glacier, northwest Greenland, from airborne radio-echo sounding (RES)^4^. The presence of ice-surface collapse basins in ice-marginal settings provided evidence for two further active subglacial lakes, recharged by surface meltwater penetrating to the bed^5–7^. The scarcity of subglacial lakes identified in Greenland has been associated with steeper ice-surface slopes, and therefore a stronger hydraulic gradient, compared to Antarctica^8,9^. However, hydrological potential calculations predict that subglacial lakes could cover ~1.2% of the GrIS bed^9^, and may constitute a significant component of the subglacial drainage system in regions conducive to their formation, such as high subglacial relief regions in the eastern sector of the ice sheet, or beneath fast-flowing outlet glaciers, such as the North East Greenland Ice Stream (NEGIS).

In contrast, over 400 subglacial lakes have been detected beneath the Antarctic Ice Sheet using a combination of ice-penetrating radar, ground-based seismic surveys and satellite surface altimetry^10^. Antarctic subglacial lakes range in size from water bodies less than 1 km in length^11^ to the largest, subglacial Lake Vostok, which is ~250 km long^12^. Hydrologically active lakes which fill and drain over decadal or shorter timescales causing uplift and subsidence of the ice surface, are typically identified from ice-surface elevation changes and commonly form close to the ice margin beneath fast-flowing outlet glaciers and ice streams^13–15^. Conversely, larger subglacial lakes, predominantly detected from RES surveys (showing bright, hydraulically flat, specular bed reflections, typically 10–20 dB stronger than surrounding bedrock), are located within 200 km of ice divides and tend to be stable over >10^3^ years^10^. Antarctic subglacial lakes have been implicated in initiating ice stream flow^16,17^ and causing transient ice accelerations driven by periodic drainage events^18–20^, highlighting their important role in ice sheet mass balance, and consequently sea-level rise^18^. Furthermore, direct sampling of subglacial lake environments has revealed complex microorganisms adapted to isolated and extreme conditions^21,22^.

In this paper, we conduct the first comprehensive survey of subglacial lakes beneath the GrIS using NASA's Operation IceBridge (OIB)  airborne RES database (1993–2016), in addition to OIB Airborne Topographic Mapper (ATM) L2 surface elevation data, and 5 m ArcticDEM composite and 2 m multi-temporal swaths to detect collapsed ice-surface basins and monitor changes in ice-surface elevation. Our results reveal a different distribution of subglacial lakes compared to their Antarctic counterparts, with a dominance of small subglacial lakes that act as a stable storage of water above the Equilibrium Line Altitude (ELA).

## Results

### Radar evidence for subglacial lakes

**Fig. 1:**
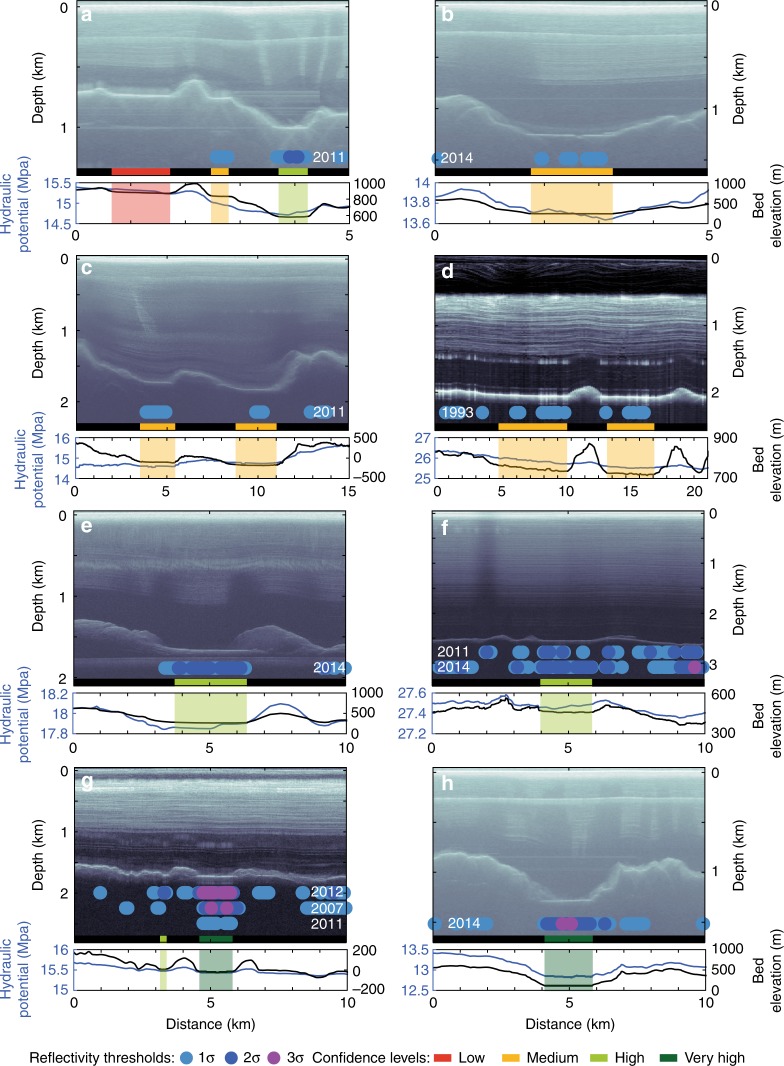
Example RES profiles for 13 subglacial lakes identified beneath the Greenland Ice Sheet in this study and the surrounding bed topography. Relative basal reflectivity values (1–3σ) are indicated by the blue-purple circles. Lakes are depicted by a bar colour-coded according to the confidence level (see Fig. 3). The bedrock elevation (black) and hydraulic potential (blue) are shown in the graphs below. **a** Lakes 32–34 along flight line 20120516_01_059, **b** lake 39 along flight line 20120503_03_037, **c** lakes 24–25 along flight line 20110329_01_019, **d** lakes 56–57 along flight line 19930702_01_012, **e** lake 29 along flight line 20140313_08_001, **f** lake 45 along flight line 20140424_01_033, with reflectivity values for years 2011 and 2014, **g** lakes 18–19 along flight line 20020530_01_007, with reflectivity values for years 2002, 2007 and 2011, **h** lakes 30–31 along flight line 20140313_08_002

Inspection of OIB airborne RES data reveals 54 previously uncharted subglacial lake candidates beneath the GrIS, accounting for 0.025% of the ~574,000 km of flight lines analysed (Supplementary Figs. 1–34). These are identified based on two methods: first, qualitative visual inspection for the presence of hydraulically flat and specular bed reflectors, analogous to subglacial lakes identified in Antarctic surveys^23,24^; second, quantitative relative basal reflectivity analysis where subglacial lakes are identified by basal reflectivity exceeding 1σ, 2σ or 3σ above the mean reflectivity within a 10 km radius surrounding region^25^ (Fig. 1). We categorise our results using four confidence levels. Low confidence where the potential lake reflector is hydraulically flat, but relative basal reflectivity anomalies are not found (Fig. 1a), or the reflector is not hydraulically flat, but exhibits reflectivity anomalies over 1σ above the mean of the surrounding region. Medium/high/very high confidence where the hydraulically flat reflector coincides with relative basal reflectivity exceeding 1σ/2σ/3σ above the mean of the surrounding region (Fig. 1b–h). Our results classify only 7% of subglacial lakes as low confidence, while 44% of the lakes identified in this study are ranked as either high or very high confidence (Supplementary Table 1). For the more uncertain lake candidates (i.e., low and medium confidence) that do not have an obvious flat reflector or with a relative basal reflectivity <2σ, we acknowledge that our approach cannot clearly differentiate very shallow lakes from flat areas of saturated sediment (akin to the fuzzy Antarctic lakes^26^).

Of the lake candidates identified using RES, 35% were surveyed multiple times between 1993 and 2016, allowing us to investigate their minimum persistence (Supplementary Table 1). Significantly, all of these subglacial lakes were detected quantitatively by high relative reflectivity indicative of basal water in each of the years surveyed, suggesting that they persisted through the period of data availability. This includes 8 lakes with RES data covering 2–5 years, 8 lakes with 13–16 years of RES data and 3 lakes that have RES data across 20 years (Supplementary Table 1). The absence of any surface elevation changes indicative of drainage or filling in both the multi-temporal OIB IceBridge L2 ATM data (2009–2017), covering 48% of RES lakes, and timestamped ArcticDEM data (2012–2016), across all RES lakes, provides further support that these lakes are stable and therefore persistent features. Together, these data provide evidence that all RES identified lakes are stable over multiple years, with 20 persisting for at least 10 consecutive years (Supplementary Table 1).

### Ice surface collapse basins

**Fig. 2:**
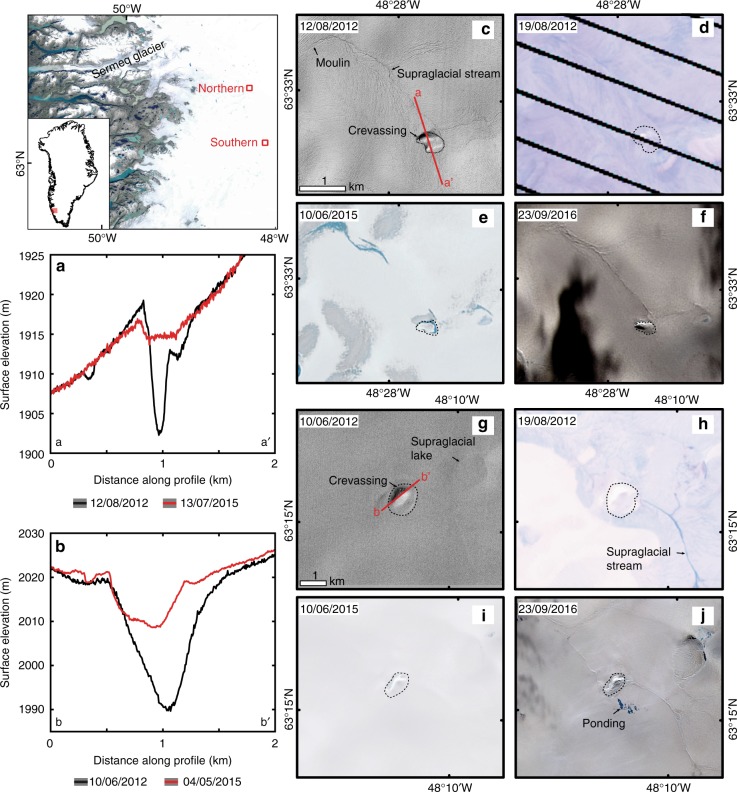
Surface elevation change and evolution of active subglacial lakes in southwest Greenland. Inset map shows location of collapse basins near Sermeq glacier. **a** Surface elevation profile across flow at northern collapse basin, measured using the ArcticDEM 2 m resolution strip files. ±0.2 m error bars are shown. **b** Surface elevation change along flow at southern collapse basin. Hillshades of the ArcticDEM and Landsat 4, 5, 6, 7 imagery showing evolution of collapse basins from depression to dome-like features through continued uplift between 2012 and 2016 for north basin (**c**–**h**) and south basin (**g**–**j**), indicating subglacial lake drainage events. Source data are provided as a Source Data file

Analysis of surface depressions with depth-to-area ratios equivalent to collapse basins previously linked to subglacial lake drainages (see methods)^6^, revealed two new surface depressions situated about 35 km apart, between Sermeq and Sioqqap Sermia glaciers in southwest Greenland (Fig. 2). The depressions measured ~0.18 and ~0.64 km^2^ in 2012 and are approximately 15.4 and 18.1 m deep, respectively (using the ArcticDEM 2 m resolution swath data). Surface elevation change, measured from multi-temporal ArcticDEM swaths, revealed ~11 ± 0.2 m uplift of the northern basin (Fig. 2a) and ~14 ± 0.2 m uplift of the southern basin between 2012 and 2015 (Fig. 2b). Landsat 4, 5, 7 and 8 imagery shows evolution from surface depressions to dome-like features through continued uplift (Fig. 2c–j). We interpret these as collapse basins that are slowly filling following subglacial lake drainage events^6^. Based on the timestamped ArcticDEM data and Landsat imagery, although equivocal, recharge at the northern collapse basin is estimated to have been occurring for >5 years, though the subglacial lake drainage event was not identified (Fig. 2a). For the southern collapse basin, a major depression appears in the Landsat imagery around 2001 and repeat imagery indicates that the depression has since shrunk in area (Fig. 2h–j), suggesting that the subglacial lake beneath has been refilling for a period of 17 years (2001–2018) so far. Observations of surface meltwater ponding, moulins (Fig. 2c) and sudden supraglacial lake drainage in this region may indicate at least partial recharge of the subglacial lake by meltwater draining to the bed^7^. No RES flight lines run directly through these lakes, therefore we cannot determine whether, like Antarctica, the radar records for these active lakes show an absence of lake reflectors^27^.

### Distribution of identified subglacial lakes

**Fig. 3:**
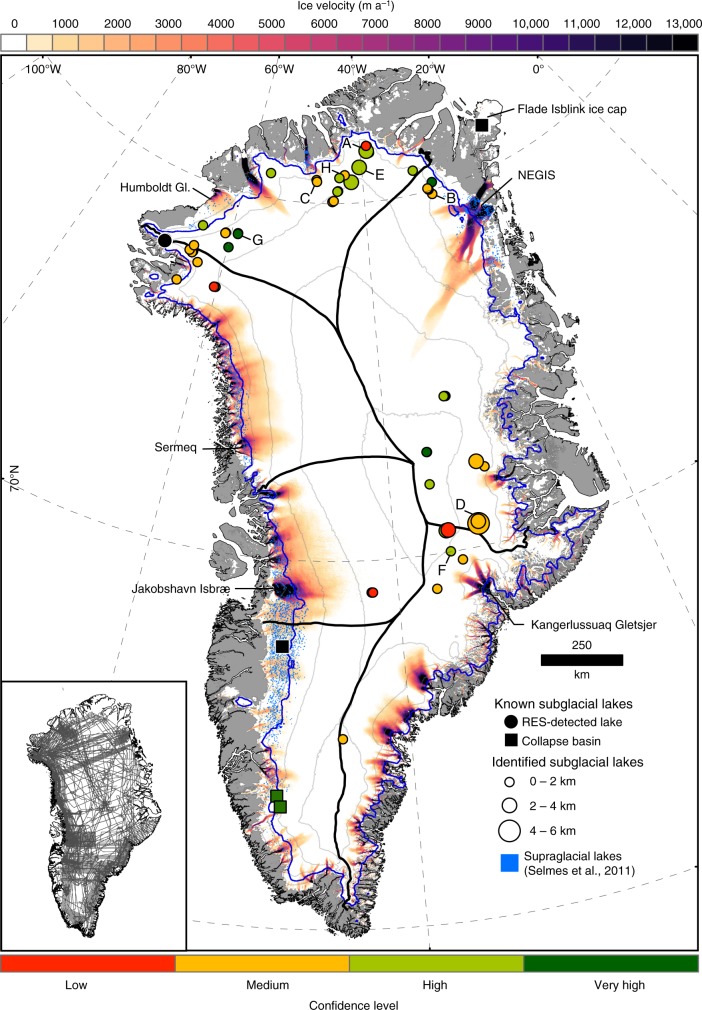
Spatial distribution of subglacial lakes identified in the radar echo sounding data. Lakes are colour-coded according to confidence level and proportionate in size to the length of the lake reflector. Black circles represent known subglacial lakes, squares depict collapse basins (black = in existing literature, green = this study). Ice-surface elevation contours at 500 m intervals are shown in grey^28^, with the equilibrium line altitude in blue (for the period 2000–2009) using MAR 3.5 forced by ERA-Interim^63^ and ice divides depicted by the thick black lines^64^. Supraglacial lake data derived from MODIS between 2005–2009 is also displayed^65^. Background map shows MEaSUREs Greenland Ice Sheet velocity map derived from InSAR^66^. Inset map displays OIB radar flight lines (1993–2016) used in this study

**Fig. 4:**
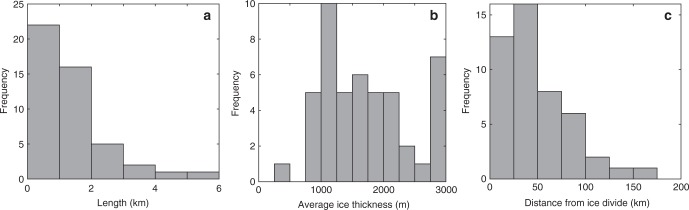
Frequency-distribution histograms of subglacial lakes identified in this study. **a** Minimum length of lake reflector identified in RES data, calculated by measuring the horizontal extent of the lake reflection. **b** Average ice thickness^28^ within 5 km^2^ area overlying the subglacial lake **c** Geodesic distance from major ice divides^64^ depicted in Fig. 3. Source data are provided as a Source Data file

The spatial distribution of subglacial lakes beneath the GrIS is illustrated in Fig. 3. We observe three main clusters of subglacial lakes in north-western, northern and central-eastern Greenland, which coincide with recent observations of ponded water from radar signal characteristics^3^. The minimum length of lake reflectors ranges from 0.2 to 5.9 km, with a mean of 1.4 km (Fig. 4a). The largest lakes (>3 km length) are located in the central-eastern sector of the ice sheet, while the smallest lakes (<0.5 km length) are predominantly situated in northwest Greenland (Fig. 3). The thickness of the ice overlying the identified subglacial lakes ranges from ~300 to 3200 m, with an average of 1647 m (Fig. 4b). Few subglacial lakes (22%) are located within 50 km of the margin, and lakes are generally absent beneath major ice divides (Fig. 4c) and fast-flowing outlet glaciers (Fig. 3). Most lakes (74%) are found beneath relatively slow-moving ice (<15 m a^–1^). Subglacial lakes appear in a variety of topographic settings; a third of lakes occur in regions of low relief confined by small bedrock bumps (≤10% bedrock gradient), while nearly a quarter of subglacial lakes are surrounded by steep bedrock hills (>30% gradient).

**Fig. 5:**
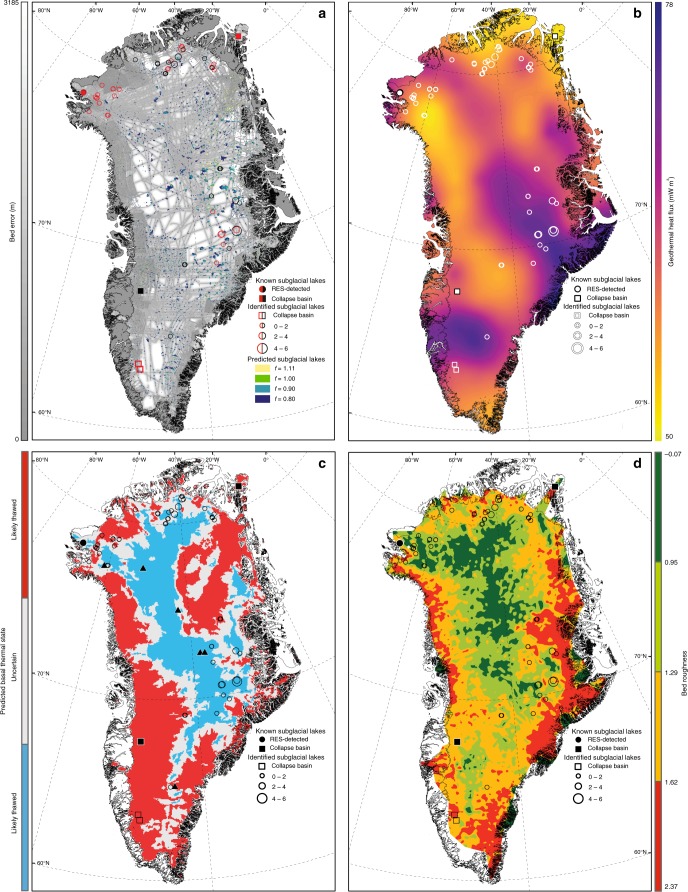
Comparison between location of identified subglacial lakes and predicted lakes, geothermal heat flux, basal thermal state and bed roughness. **a** Predicted subglacial lake locations, using Shreve hydraulic potential equation and varying flotation fractions (*f*) overlain on the BedMachine DEM error map^28^. Lakes in black coincide with hydraulic minima (using *f* = 0.9), whilst those in red were not successfully predicted. **b** Estimated geothermal heat flux derived from magnetic data^29^. **c** Predicted basal thermal state^1^ (blue = likely frozen, white = uncertain, red = likely thawed). **d** Dimensionless bed roughness beneath the GrIS^33^, measured using the fast Fourier transform approach which converts bed topography (derived from radar echo sounding) into the frequency domain. Known subglacial lakes in existing literature are shown by black circles (RES-detected) and squares (detected through elevation changes). Deep ice core locations are depicted by black triangles. Lakes identified in this study are shown by circles, and the size of the circle is proportional to the minimum length of the lake reflector

The spatial distribution of simulated subglacial lakes beneath the GrIS, following hydraulic potential analysis methods^9^, updated for BedMachine v3^28^ (150 m resolution) is shown in Fig. 5a. Only 32% of our identified subglacial lakes are located within 1 km of estimated hydraulic minima (*f* = 0.9). This is reduced to 9% when a flotation fraction of *f* = 1 is applied. The majority of subglacial lakes coincide with regions of estimated geothermal heat flux^29^ between 50 and 61 mW m^−2^ (Fig. 5b), while central-eastern lakes mostly appear in the zone of elevated geothermal heat flux (>66 mW m^−2^). Figure 5c displays locations of identified subglacial lakes in relation to basal thermal state predictions, estimated using thermomechanical ice flow modelling, radiostratigraphy, surface ice velocity and borehole observations^1^. 20% of subglacial lakes identified in the RES data are situated in the likely frozen region, all of which are found in central-eastern Greenland, a quarter of lakes are located in the likely thawed region, predominantly in north-western Greenland, and the remaining 55% are detected in regions where the predicted basal thermal state is uncertain (Fig. 5c). The majority (63%) of subglacial lakes in this study are found in regions of relatively high (dimensionless) bed roughness (>1.29), towards the margins (Fig. 5d).

## Discussion

The subglacial lake candidates identified in this study differ from those observed in Antarctica, both in terms of topographic setting and size. Antarctic subglacial lakes detected by RES typically occur in close proximity to ice divides under thick (>4000 m), warm-based ice^30^, with the largest subglacial lakes occupying tectonically controlled topographic depressions^31,32^. In contrast, Greenland subglacial lakes are typically absent beneath the isostatically depressed, smooth interior basin along the main N–S ice divide, and are instead concentrated toward the ice margin.  In the centre of the GrIS, ice is largely frozen to its bed^1^, with water becoming more prevalent towards the margin where ice surface speeds are typically higher^2^ and surface-to-bed hydraulic connectivity more likely. This is in contrast, however, to a recent analysis of radar signal characteristics which suggests extensive ponded water around the North GRIP drill site at the onset of the NEGIS^3^. The largest GrIS subglacial lakes are constrained by steep bedrock relief in the East Greenland subglacial mountain chain, whereas the smaller lakes (<2 km) tend to be prevalent in regions associated with low subglacial roughness, such as northwest Greenland^33^. On average, GrIS subglacial lakes are nearly eight times shorter than their Antarctic counterparts (1.4 km compared to 11 km on average in Antarctica, excluding Lake Vostok^34^), reflecting the steeper average ice-surface (and thus subglacial hydraulic) gradient and different bed topographic settings controlling the locations of subglacial lakes. In particular, Greenland bed topography is relatively flat and well-organised with respect to ice flow^28^, whereas the topography beneath Antarctica is more complex^35^, and therefore offers sites where large volumes of basal water can be stored e.g., Subglacial Lake Vostok, Concordia and Ellsworth.

About 40% of identified subglacial lakes in Antarctica are active (i.e., evidence of drainage/filling from ice-surface elevation changes), and these are mostly found beneath ice streams in West Antarctica^36^. In contrast, we observe only two further examples of active subglacial lakes in Greenland (4 in total; 6.7% of all discovered lakes), and a general absence of subglacial lakes beneath fast-flowing outlet glaciers. This could be due to the reliance on altimetry methods rather than RES techniques to resolve active lakes^36^. However, as well as assessing surface elevation changes above RES-detected lakes using both timestamped ArcticDEM and OIB IceBridge ATM2 datasets, we also carried out an ice-sheet wide survey for ice surface collapsed basins using the 5 m resolution ArcticDEM v2.0 mosaic^37^ (see methods), finding just two collapsed ice basins consistent with lake drainage events (Fig. 2).

There is a general paucity of lakes in fast-flowing western and southern sectors of the GrIS, and beneath the NEGIS (Fig. 3), despite hydraulic potential^9^ and bed reflectivity analysis^2,3^ predicting widespread basal water in these regions. The southern and western sectors of the GrIS have extensive ablation areas where large volumes of surface meltwater are generated each summer^38,39^. Lakes in the ablation area may be more difficult to detect from airborne radar because the ice is thin, and the ice-surface is rougher and steeper than further inland. In addition, we hypothesise that the drainage of this meltwater to the bed inhibits subglacial lake formation on annual or longer timescales, due to the seasonal evolution of efficient subglacial drainage systems able to connect and drain stored water to the ice margin^40^. Evidence of seasonal subglacial water storage from RES data supports this; water is stored on bedrock plateaus during the winter and flushed out during summer when efficient subglacial drainage develops^41^. These small, seasonally active subglacial lakes are difficult to resolve from monitoring ice-surface elevation alone due to large seasonal mass changes in the ablation zone, including the drainage and filling of supraglacial lakes, which can coincide with subglacial lake locations^42^. Over longer time-scales, focused erosion by subglacial water cutting channels into the bed beneath fast-flowing southern and western sectors (e.g., due to the perennial drainage of supraglacial lakes) and the NEGIS (e.g., due to geothermal and frictional basal melting) may lead to the removal of hydraulic minima^43,44^. This may explain why the few active subglacial lakes large and stable enough to be detected under the GrIS are found in close proximity to the ELA, where there is less surface melting and where the formation of efficient subglacial drainage is inhibited by thicker ice and low surface slopes^45,46^. More persistent, RES-detected subglacial lakes, are associated with regions of low melt input variability; the majority of subglacial water recharging these lakes is generated from elevated geothermal heat flux rather than surface melt (the region of surface lakes only coincides with one RES detected subglacial lake–Fig. 3)^29^. Finally, the lack of subglacial lakes detected beneath the fast-flowing southern and western sectors of the GrIS and the NEGIS may represent a limitation of our approach. In particular, we consistently struggle to discriminate lakes in uniformly thawed regions^1^ (Fig. 5c), where they are predicted to be prevalent^9^, likely because extensive water/saturated sediment results in a higher mean reflectivity and thus reduced relative contrast of the lake compared to background bed conditions. This may partially explain the discrepancy with previous studies which find extensive ponded water at the onset of and beneath the NEGIS^3^.

The recall of just 32% of the identified subglacial lakes by hydraulic potential analysis using a flotation fraction of *f* = 0.9 (Fig. 5a) contrasts with similar analyses in Antarctica that successfully recall >50% of the known subglacial lakes^9,47^. We suggest three reasons why hydraulic potential analysis is less useful at predicting subglacial lakes in Greenland, even under steady state basal conditions. Firstly, there is a clear size bias, with larger lakes being generally easier to recall^9^. As Greenland lakes are on average 8 times shorter than those in  Antarctica, they are typically more difficult to discern from the hydraulic potential analysis. However, recall of subglacial lakes is still low even in regions of the ice sheet characterised by high data coverage and low bed error, such as northwest Greenland (Fig. 5a), where we would expect smaller hydraulic minima to be more accurately constrained. We therefore posit that low bed roughness in areas such as northwest Greenland^33,48^ (Fig. 5d), makes it more difficult to accurately pick out hydraulic minima because there is a small difference in elevation between the lake surface and basin lip (0% recall in northwest Greenland using a flotation fraction of *f* = 0.9). In contrast, regions with higher bed roughness, such as north-eastern Greenland^33^ have correspondingly higher recall rates (44%), and indeed, like Antarctica, lakes that form in deep topographic depressions also tend to be larger. Finally, only 25% of low and 31% of medium confidence lake candidates fall within 1 km of hydropotential lows, compared to 50% for very high confidence lakes, supporting the inference that these lower confidence lakes may actually comprise regions of swampy saturated sediment rather than well-defined lake basins^26^.

We have shown that subglacial lakes are more prevalent in Greenland than previously assumed. These subglacial lake candidates are repeatedly identified in RES data, suggesting that they could act as long-term meltwater reservoirs; active drainage of lakes towards the ice sheet margin is restricted or difficult to detect. The majority of subglacial lakes are concentrated in the uncertain regions of predicted basal thermal state^1^ where ice sheet models and ice-penetrating radar do not agree on whether the bed is frozen or thawed. As our method is based on relative bed echo strength, it is likely to pick out lakes surrounded by colder, less reflective bed material more easily, compared to lakes surrounded by warm-bedded regions (e.g., beneath the NEGIS). Thus, our results may indicate that this uncertain region is heterogenous (a mosaic of cold and warm basal conditions) on length scales that enable us to detect multiple water pockets from RES, but which ice-sheet models running at coarser resolution would have difficulty resolving. The lack of alignment between RES-detected lakes and hydraulic potential analyses may therefore occur because subglacial water is spatially constrained by the prevalence of frozen bed conditions rather than topography^43^. The presence of subglacial lakes scattered within these uncertain regions consequently helps to further constrain the thermal state of the bed.

Similar to previous investigations^4–7^ we suggest that the actively draining lakes in southwest Greenland are recharged by seasonal surface meltwater transferred to the bed due to the association of these locations with surface meltwater ponding and drainage. Although further study is required to assess the net influence of subglacial lakes on GrIS dynamics, it is likely to be limited due to the paucity of active lakes large enough to induce dynamic surface height changes, their proximity to the margin, and the strong control of surface meltwater in determining the character of subglacial drainage and its influence on ice-sheet dynamics. However, this inventory of Greenland subglacial lakes could be utilised to locate candidates for direct sampling. Seasonal surface meltwater transporting microbial life, minerals, organic matter and pollutants into the hydraulically connected subglacial system may have important implications for basal biogeochemistry, while their sedimentary deposits contain an archive of ice sheet evolution^49^ and palaeoenvironmental change^50^.

**Fig. 6:**
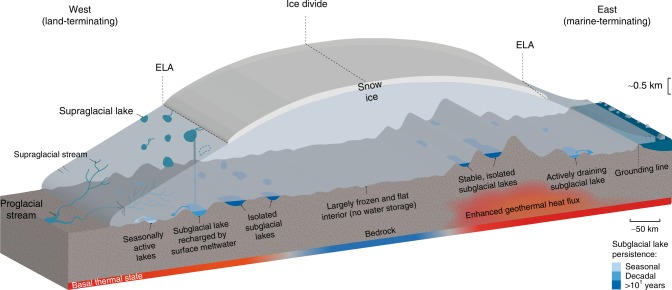
Conceptual model of Greenland ice sheet hydrological system, illustrating supraglacial and subglacial features. Subglacial lakes are colour-coded according to lake persistence, those that are less distinct and could represent shallow water lenses or patches of saturated sediment are coloured brown. Estimated basal thermal state is represented by the red to blue colour bar, with red being likely thawed, white being uncertain regions, and blue being likely frozen^1^. In the ice sheet interior, the ice is mostly frozen to the bed and the basal topography is flat, prohibiting water storage and subglacial lake formation. Above the equilibrium line altitude (ELA) but away from ice divides, where the bed is predicted to comprise a heterogeneous patchwork of frozen and warm-bedded conditions, subglacial lakes are relatively common but stable features. Around the ELA, subglacial lakes are hydrologically active and recharged seasonally by inputs of surface water. Finally, relatively numerous small subglacial lakes that drain on seasonal timescales when efficient subglacial networks develop during the melt season likely exist in the ablation zone

There is little doubt that our inventory, although representing a significant augmentation of the number of identified lakes, is incomplete and further subglacial lakes remain to be discovered with continued expansion and repetition of OIB flights, in addition to the launch of NASA’s IceSat-2 satellite enabling improved active subglacial lake identification. However, the distribution (Fig. 3), size (Fig. 4a) and activity (Supplementary Table 1) of identified subglacial lakes allows us to propose a conceptual model of GrIS subglacial water storage and dynamics (Fig. 6). Beneath the ice sheet interior there is limited detectable water storage due to the frozen and flat bed. Where the bed is inferred to comprise a heterogeneous patchwork of frozen and warm-bedded conditions, isolated subglacial lakes persist from year to year. Regions characterised by high relief basal topography and geothermal heat flux sustain the largest lakes (e.g., large parts of East Greenland). We identify a range of lake detection confidence levels, with some lakes clearly distinguishable, whereas others are less distinct and may represent shallow water lenses or even patches of saturated sediment. Around the ELA, there is some evidence of hydrologically active lakes recharged seasonally by inputs of surface water^6^. However, relatively numerous small subglacial lakes that drain on seasonal timescales when efficient subglacial networks develop during the melt season, likely exist in the ablation zone^41^. These lakes are difficult to detect from monitoring surface elevation due to their small size and the large seasonal surface mass changes in the ablation zone. Although we do not identify many subglacial lakes beneath fast-flowing warm-based regions of the ice-sheet, we do not rule out significant additional water storage here given the potential limitations of our approach for effectively identifying lakes where the mean reflectivity is high. Long-term basal meltwater storage beneath the region beyond the ELA could be activated in the future as the ablation area migrates inland^51^. The resulting increased input of meltwater to the bed at higher elevations could open new subglacial drainage pathways through enhanced sliding and potentially connect this dormant storage to the ice sheet margin.

## Method

### Subglacial lake identification from radio-echo sounding

We analysed OIB airborne RES profiles collected between 1993 and 2016 and obtained from the Center for Remote Sensing of Ice Sheets (CReSIS) archive (http://data.cresis.ku.edu)^52^. A number of RES instruments (Improved Coherent Radar Depth Sounder (ICORDS), Multi-Channel Radar Depth Sounder (MCRDS), and Multi-Channel Coherent Radar Depth Sounder (MCoRDS)) on board various NASA aircraft were used, which have a frequency range of 140–230 MHz and a transmit power of 200–2000 W. We used the L1B synthetic aperture radar (SAR) products for our analysis, which were processed by CReSIS. The basic processing steps included pulse compression with time and frequency windowing, followed by compensation for aircraft motions. The data were then stacked coherently before application of focused SAR processing. The depth range resolution in ice after the final processing was ~4.3 m with a final product along-track resolution of ~25 m. Bed depths were defined by the returned bed echoes identified by CReSIS with automatic detectors and manual pickers.

We interrogated over 574,000 km of radar echograms for subglacial lakes based on two metrics: first, qualitative visual inspections for hydraulically flat and smooth bed reflectors^23,24,26^, second, quantitative analysis of bed reflectivity^53^. Relative basal reflectivity, determined from the bed returned power, is a well-established method for detecting subglacial water and basal conditions beneath both the Antarctic Ice Sheet^53–59^ and the GrIS^25,41,44^. Studies typically suggest anomalies of 10–20 dB constitute a suitable threshold for distinguishing wet and dry beds, however, this threshold is sensitive to basal roughness; high reflectivity anomalies can also be associated with a smooth, flat bed and saturated sediment^48^. We therefore use relative basal reflectivity thresholds based on the statistics of the bed returned power within a 10 km radius around the identified lake (1σ, 2σ and 3σ from the mean), together with hydraulically flat reflectors at the ice-bed interface, to delineate subglacial lakes. Following the qualitative identification of subglacial lakes in the RES data, lakes were classified based on the coincidence of horizontal reflectors and high relative basal reflectivity. Confidence levels range from low confidence (a flat reflector but low relative reflectivity) to very high confidence (a flat reflector which is three standard deviations above the mean).

### Surface collapse basin identification

In addition to RES analysis, we applied a simple technique to identify potential collapse basins in the surface of the ice sheet, which are indicative of subglacial lake drainage and recharge events^6,7^. We removed the sinks in the high resolution (5 m) ArcticDEM (v2.0) from the Polar Geospatial Center^37^ and then subtracted this from the original DEM to identify topographic surface depressions. These ice-surface depressions were then classified based on the similarity of their depth-to-area ratios to existing collapse basins (21.07 m to 0.55 km^2^). Multi-temporal ArcticDEM strip files (2012–2016) at 2 m resolution enabled elevation change detection, providing additional information about potential subglacial lake drainage events.

### Surface elevation change measurements using Operation IceBridge ATM

OIB Airborne Topographic Mapper (ATM) Level-2 Icessn Elevation, Slope and Roughness version 2 dataset, provided and processed by National Snow and Ice Data Center (NSIDC)^60^, were used to assess the activity of subglacial lakes detected in the RES data. This laser altimeter system provides swath surface elevation measurements at a sampling frequency of 5 kHz, cross-track width of ~400 m, and a footprint diameter of ~1 m. Co-located measurements are repeated annually (2009–2017), providing a surface elevation time series for each identified subglacial lake.

### Hydraulic potential analysis

To calculate hydraulic potential gradients, we apply the Shreve^61^ hydraulic potential equation using BedMachine v3^28^ (150 m resolution) bed elevation and ice thickness data, following methods outlined in a previous study^9^. Minima in the hydraulic potential surface were identified using TopoToolbox^62^, a Matlab-based software. Hydraulic potential surfaces were calculated for a range of flotation fraction values from 0.8 to 1.11 to test the sensitivity of lake locations to changes in water pressure.

## Supplementary information

Supplementary Information

Description of Additional Supplementary Files

Supplementary Data 1

Source Data

## Data Availability

Radar echo sounding data are freely available from CReSIS/ NASA Operation IceBridge and accessed via the National Snow and Ice Data Center (NSIDC) archive (https://data.cresis.ku.edu/data/rds/). MEaSUReS InSAR ice velocity measurements were also accessed from NSIDC via 10.5067/IAGYM8Q26QRE. NASA Operation IceBridge BedMachine Greenland v3 ice thickness and surface data are available through NSIDC at http://nsidc.org/data/IDBMG4. DEMs provided by the Polar Geospatial Center under NSF OPP awards 1043681, 1559691 and 1542736. The Landsat images used for this work are available from the US Geological Survey via http://earthexplorer.usgs.gov/. Predicted basal thermal state of the Greenland Ice Sheet data are freely available from the NSIDC Distributed Active Archive Centre at https://nsidc.org/data/RDBTS4.

## Code availability

The data that support the findings of this study are available from the corresponding author upon request.
