# Supplementary material for: Distribution and dynamics of Greenland subglacial lakes
Source: Nat Commun. 2019 Jun 26;10:2810. doi: 10.1038/s41467-019-10821-w (PMC6594964; doi:10.1038/s41467-019-10821-w)
Supplement: Supplementary file 1 — Supplementary Information [file 41467_2019_10821_MOESM1_ESM.pdf]

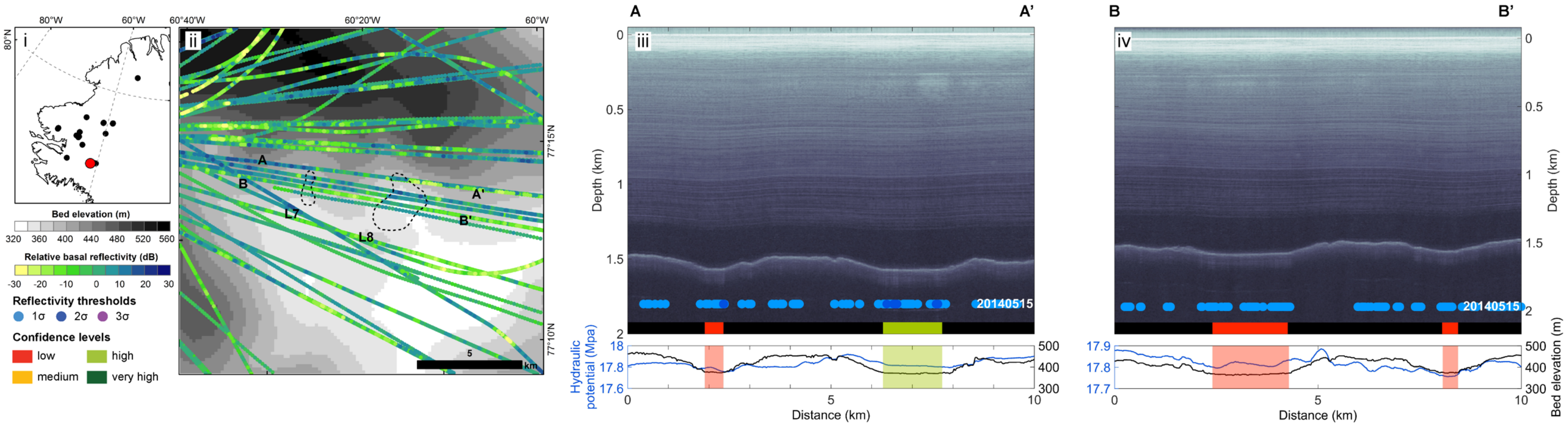

Supplementary Figure 1: Radar evidence for Greenland subglacial lakes. (i) Inset map showing location of subglacial lake L7 and L8 (red) and neighbouring lakes found in this study (black). (ii) Bed topography of the region with relative basal reflectivity along Operation IceBridge flight paths. Estimated lake extent is shown by the dashed line. Radar profile along transect (iii) A-A' (20140515_02_005) and (iv) B-B' (20145015_02_068). Subglacial lakes are depicted by a bar colour-coded according to the confidence level. Relative basal reflectivity thresholds, based on the statistics of the bed returned power within 20 km of the identified lake (1-3 σ from the mean), are indicated by the blue-purple circles. Lower graphs show bedrock elevation (black) and hydraulic potential (blue).

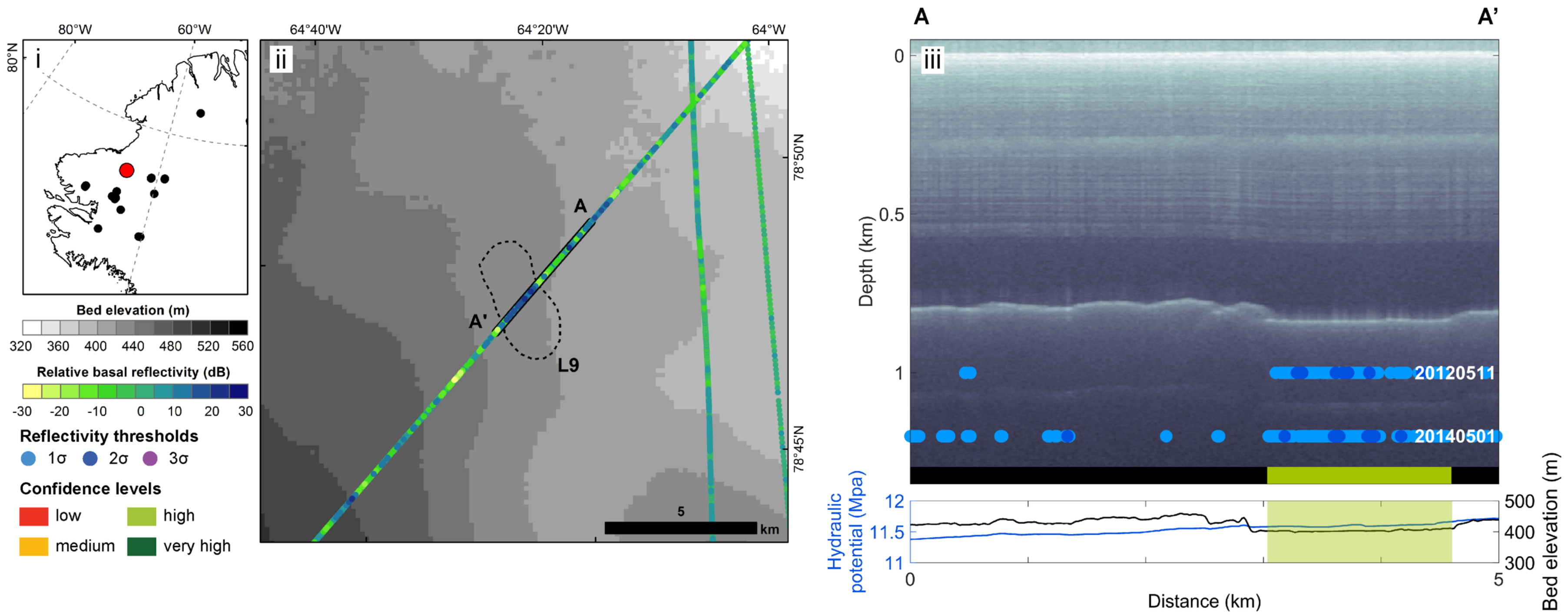

Supplementary Figure 2: Radar evidence for Greenland subglacial lakes. (i) Inset map showing location of subglacial lake L9 (red) and neighbouring lakes found in this study (black). (ii) Bed topography of the region with relative basal reflectivity along Operation IceBridge flight paths. Estimated lake extent is shown by the dashed line. Radar profile along transect (iii) A-A' (20120511_01_028/ 20140501_01_029). Subglacial lakes are depicted by a colour-coded according to the confidence level. Relative basal reflectivity thresholds, based on the statistics of the bed returned power within 20 km of the identified lake (1-3 σ from the mean), are indicated by the blue-purple circles. Lower graphs show bedrock elevation (black) and hydraulic potential (blue).

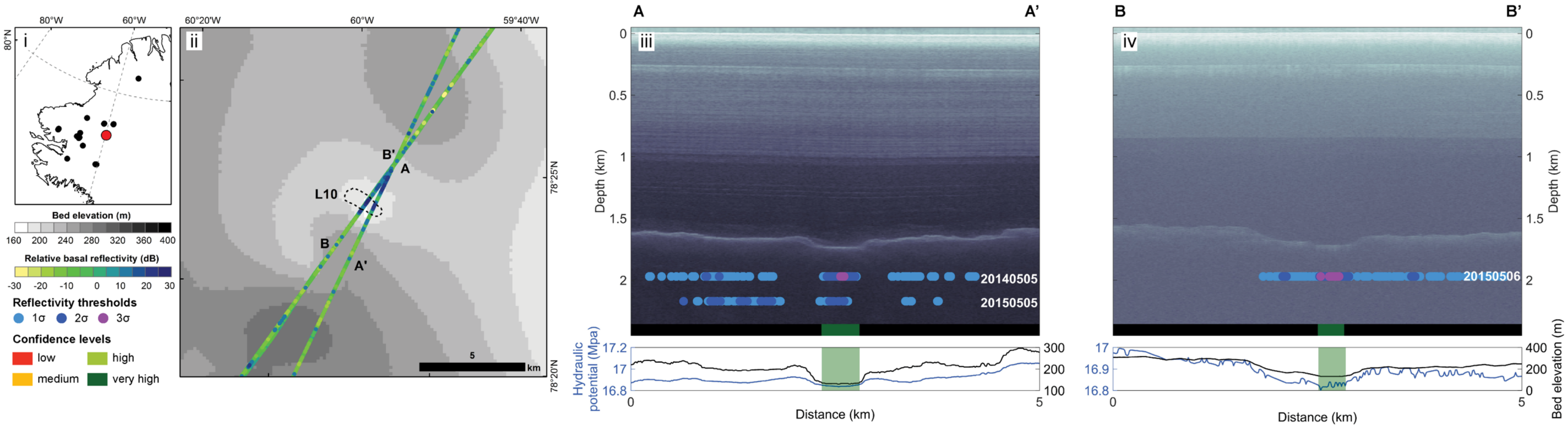

Supplementary Figure 3: Radar evidence for Greenland subglacial lakes. (i) Inset map showing location of subglacial lake L10 (red) and neighbouring lakes found in this study (black). (ii) Bed topography of the region with relative basal reflectivity along Operation IceBridge flight paths. Estimated lake extent is shown by the dashed line. Radar profile along transect (iii) A-A' (20140505_01_042/ 20150505_02_042) and (iv) B-B' (20150506_02_008). Subglacial lakes are depicted by a bar colour-coded according to the confidence level. Relative basal reflectivity thresholds, based on the statistics of the bed returned power within 20 km of the identified lake (1-3 σ from the mean), are indicated by the blue-purple circles. Lower graphs show bedrock elevation (black) and hydraulic potential (blue).

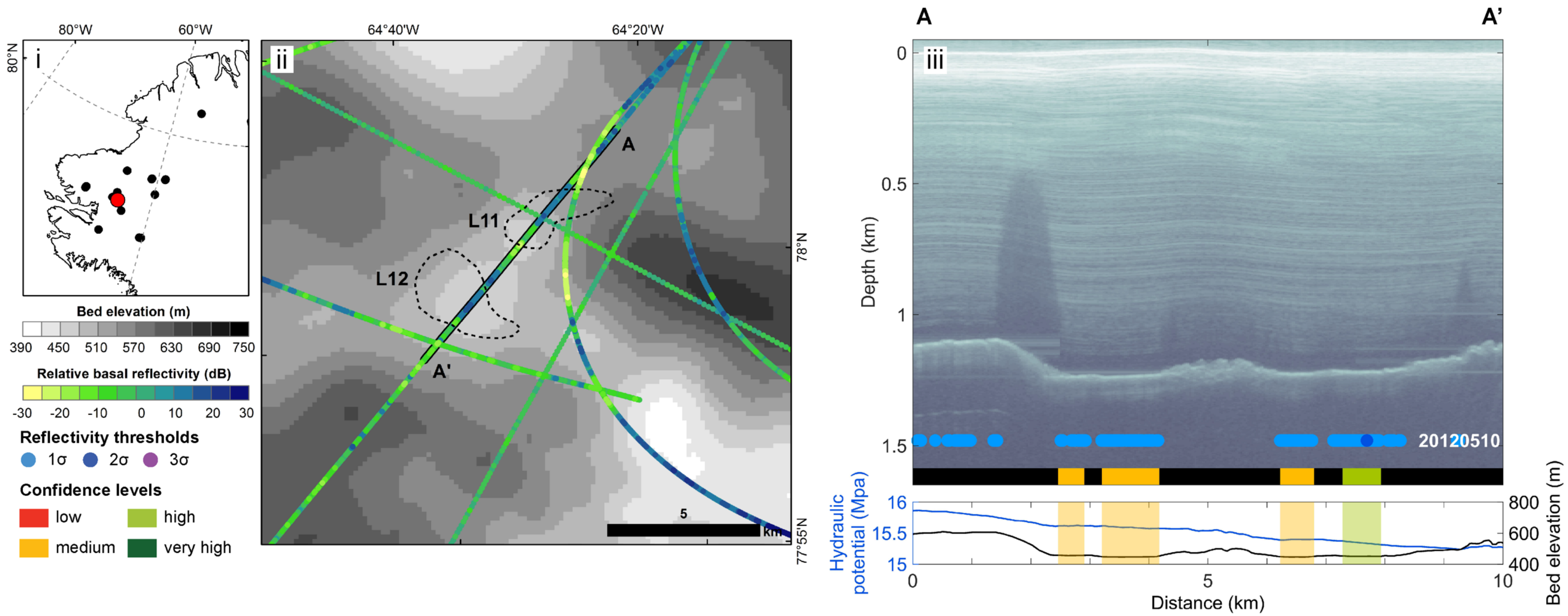

Supplementary Figure 4: Radar evidence for Greenland subglacial lakes. (i) Inset map showing location of subglacial lake L11 and L12 (red) and neighbouring lakes found in this study (black). (ii) Bed topography of the region with relative basal reflectivity along Operation IceBridge flight paths. Estimated lake extent is shown by the dashed line. Radar profile along transect (iii) A-A' (20120510_01_056). Subglacial lakes are depicted by a bar colour-coded according to the confidence level. Relative basal reflectivity thresholds, based on the statistics of the bed returned power within 20 km of the identified lake (1-3 σ from the mean), are indicated by the blue-purple circles. Lower graphs show bedrock elevation (black) and hydraulic potential (blue).

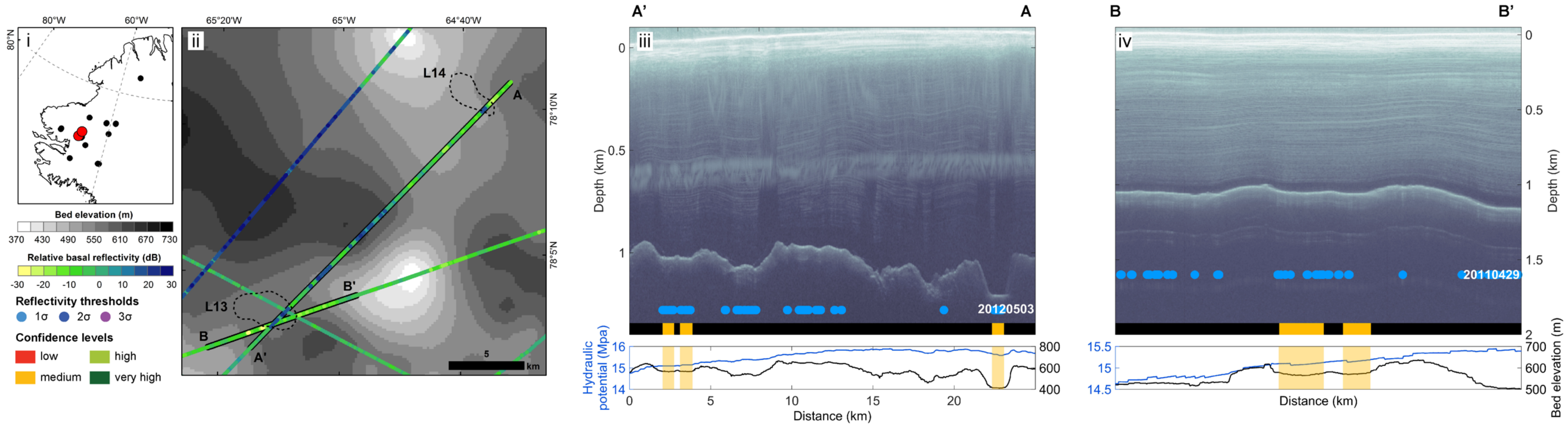

Supplementary Figure 5: Radar evidence for Greenland subglacial lakes. (i) Inset map showing location of subglacial lake L13 and L14 (red) and neighbouring lakes found in this study (black). (ii) Bed topography of the region with relative basal reflectivity along Operation IceBridge flight paths. Estimated lake extent is shown by the dashed line. Radar profile along transect (iii) A-A' (20120503_01_002) and (iv) B-B' (20110429_01_005). Subglacial lakes are depicted by a bar colour-coded according to the confidence level. Relative basal reflectivity thresholds, based on the statistics of the bed returned power within 20 km of the identified lake (1-3 σ from the mean), are indicated by the blue-purple circles. Lower graphs show bedrock elevation (black) and hydraulic potential (blue).

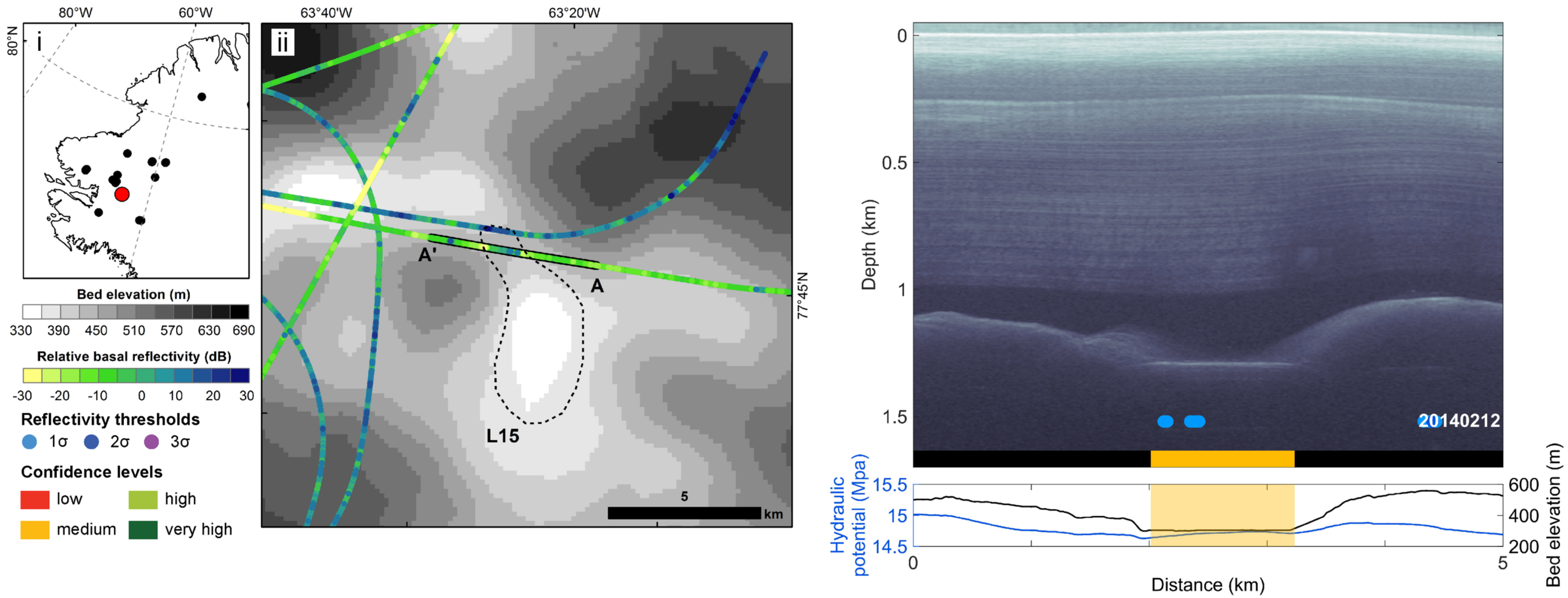

Supplementary Figure 6: Radar evidence for Greenland subglacial lakes. (i) Inset map showing location of subglacial lake L15 (red) and neighbouring lakes found in this study (black). (ii) Bed topography of the region with relative basal reflectivity along Operation IceBridge flight paths. Estimated lake extent is shown by the dashed line. Radar profile along transect (iii) A-A' (20140512_01_025). Subglacial lakes are depicted by a bar colour-coded according to the confidence level. Relative basal reflectivity thresholds, based on the statistics of the bed returned power within 20 km of the identified lake (1-3 σ from the mean), are indicated by the blue-purple circles. Lower graphs show bedrock elevation (black) and hydraulic potential (blue).

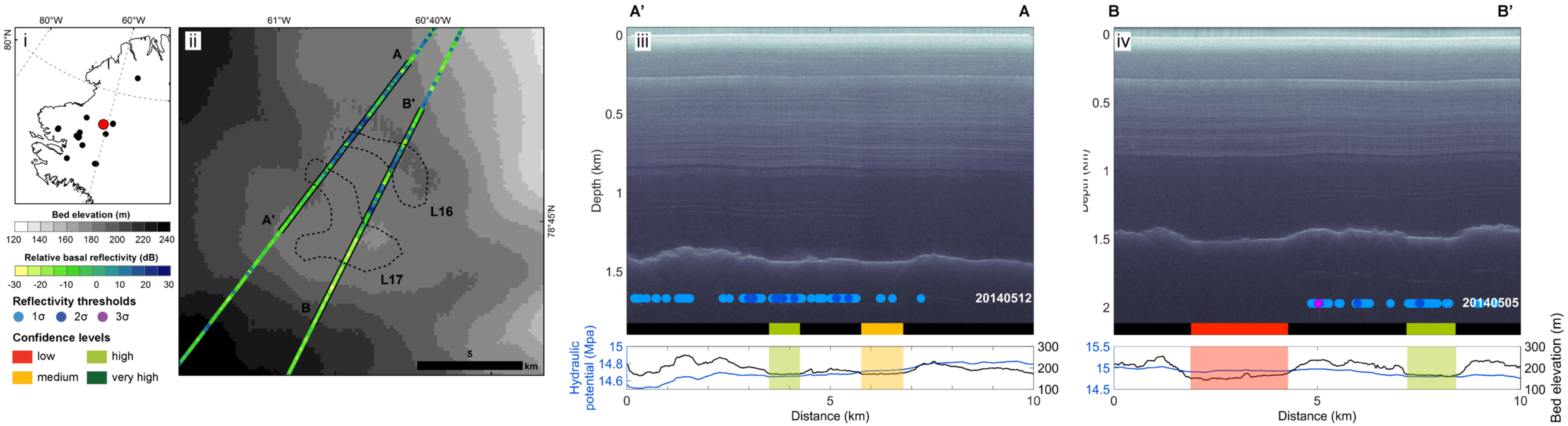

Supplementary Figure 7: Radar evidence for Greenland subglacial lakes. (i) Inset map showing location of subglacial lake L16 and L17 (red) and neighbouring lakes found in this study (black). (ii) Bed topography of the region with relative basal reflectivity along Operation IceBridge flight paths. Estimated lake extent is shown by the dashed line. Radar profile along transect (iii) A-A' (20140512_01_022) and (iv) B-B' (20140505_01_032). Subglacial lakes are depicted by a bar colour-coded according to the confidence level. Relative basal reflectivity thresholds, based on the statistics of the bed returned power within 20 km of the identified lake (1-3 σ from the mean), are indicated by the blue-purple circles. Lower graphs show bedrock elevation (black) and hydraulic potential (blue).

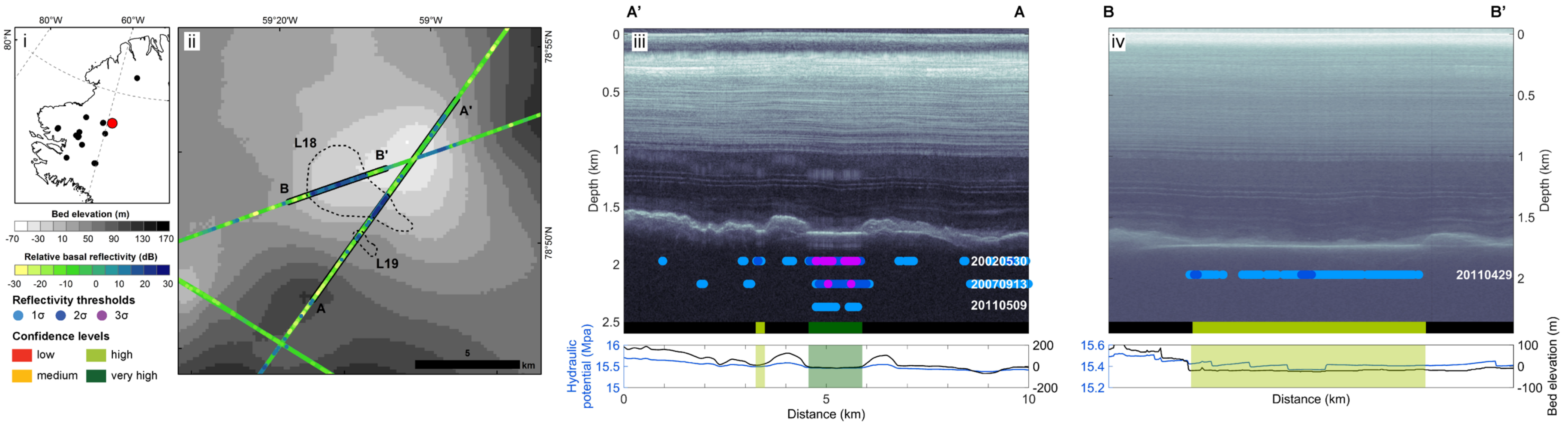

Supplementary Figure 8: Radar evidence for Greenland subglacial lakes. (i) Inset map showing location of subglacial lake L18 and L19 (red) and neighbouring lakes found in this study (black). (ii) Bed topography of the region with relative basal reflectivity along Operation IceBridge flight paths. Estimated lake extent is shown by the dashed line. Radar profile along transect (iii) A-A' (20020530_01_007/ 20070913_02_005/ 20110509_01_008) and (iv) B-B' (20110429_01_008). Subglacial lakes are depicted by a bar colour-coded according to the confidence level. Relative basal reflectivity thresholds, based on the statistics of the bed returned power within 20 km of the identified lake (1-3 σ from the mean), are indicated by the blue-purple circles. Lower graphs show bedrock elevation (black) and hydraulic potential (blue).

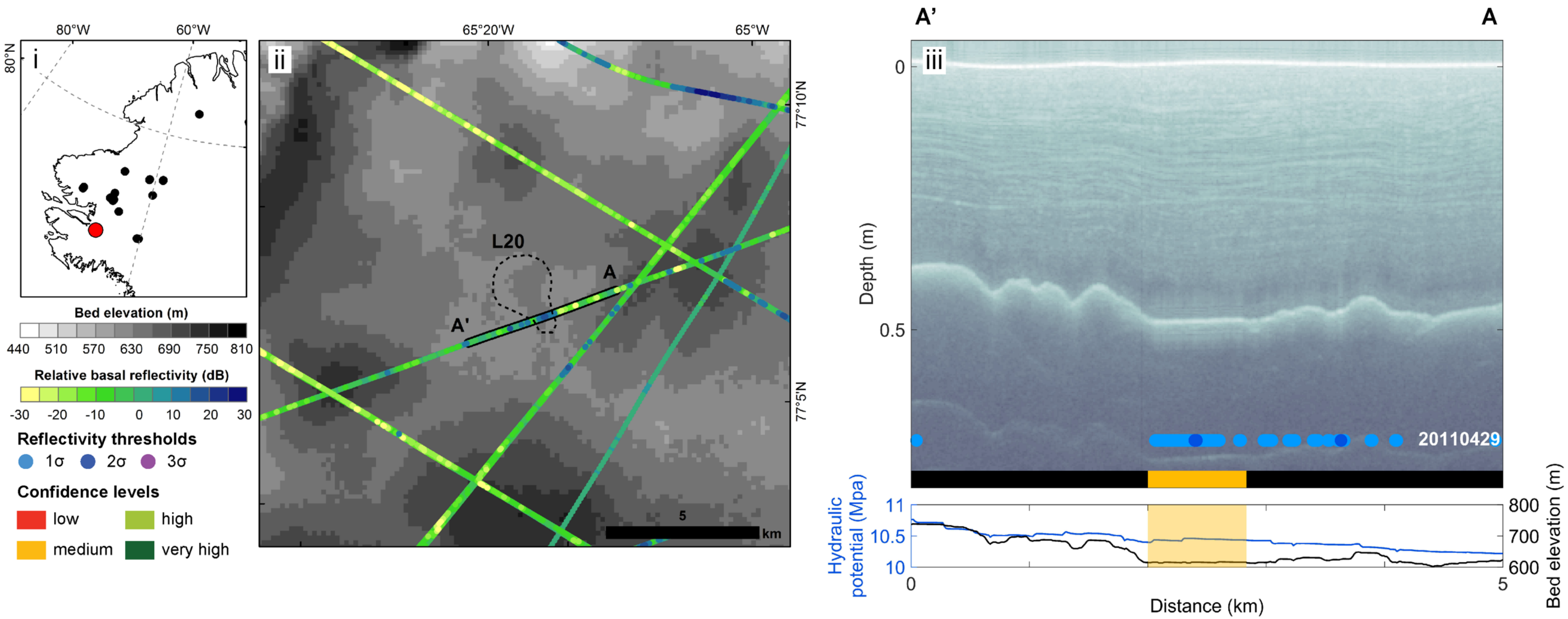

Supplementary Figure 9: Radar evidence for Greenland subglacial lakes. (i) Inset map showing location of subglacial lake L20 (red) and neighbouring lakes found in this study (black). (ii) Bed topography of the region with relative basal reflectivity along Operation IceBridge flight paths. Estimated lake extent is shown by the dashed line. Radar profile along transect (iii) A-A' (20110429_02_028). Subglacial lakes are depicted by a bar colour-coded according to the confidence level. Relative basal reflectivity thresholds, based on the statistics of the bed returned power within 20 km of the identified lake (1-3 σ from the mean), are indicated by the blue-purple circles. Lower graphs show bedrock elevation (black) and hydraulic potential (blue).

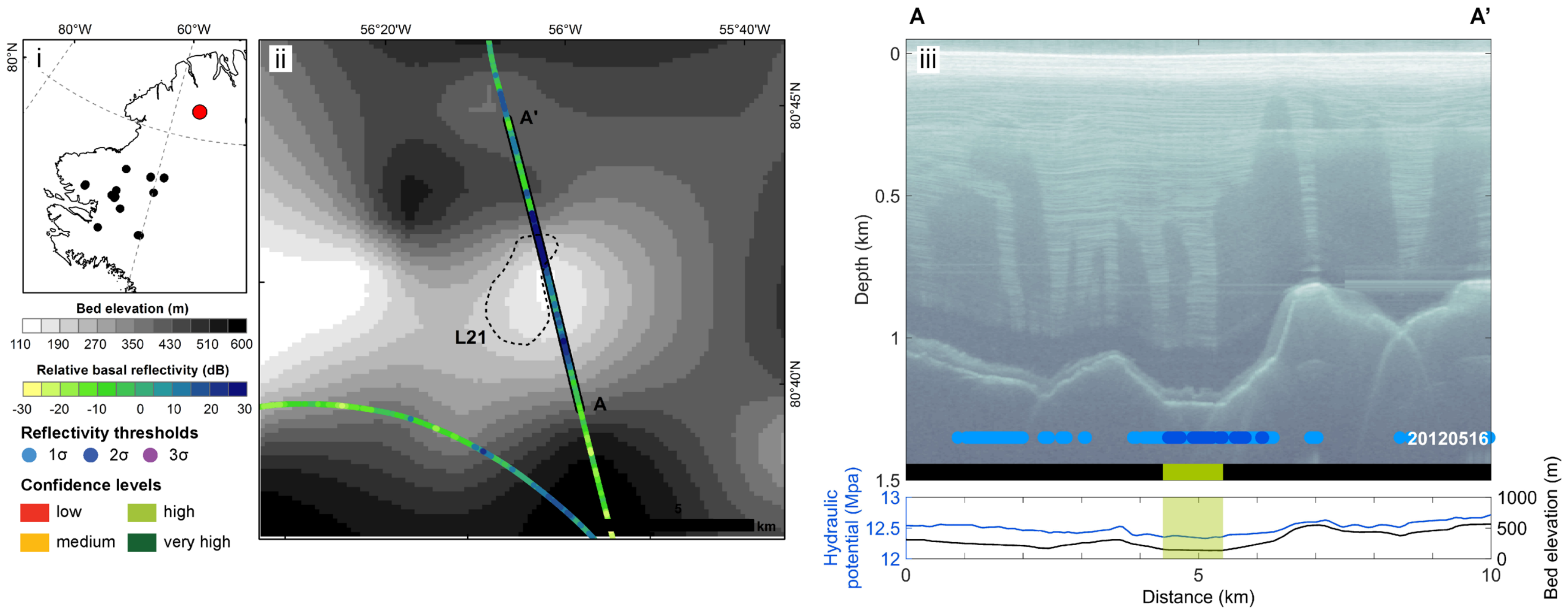

Supplementary Figure 10: Radar evidence for Greenland subglacial lakes. (i) Inset map showing location of subglacial lake L21 (red) and neighbouring lakes found in this study (black). (ii) Bed topography of the region with relative basal reflectivity along Operation IceBridge flight paths. Estimated lake extent is shown by the dashed line. Radar profile along transect (iii) A-A' (20120516_01_018). Subglacial lakes are depicted by a bar colour-coded according to the confidence level. Relative basal reflectivity thresholds, based on the statistics of the bed returned power within 20 km of the identified lake (1-3 σ from the mean), are indicated by the blue-purple circles. Lower graphs show bedrock elevation (black) and hydraulic potential (blue).

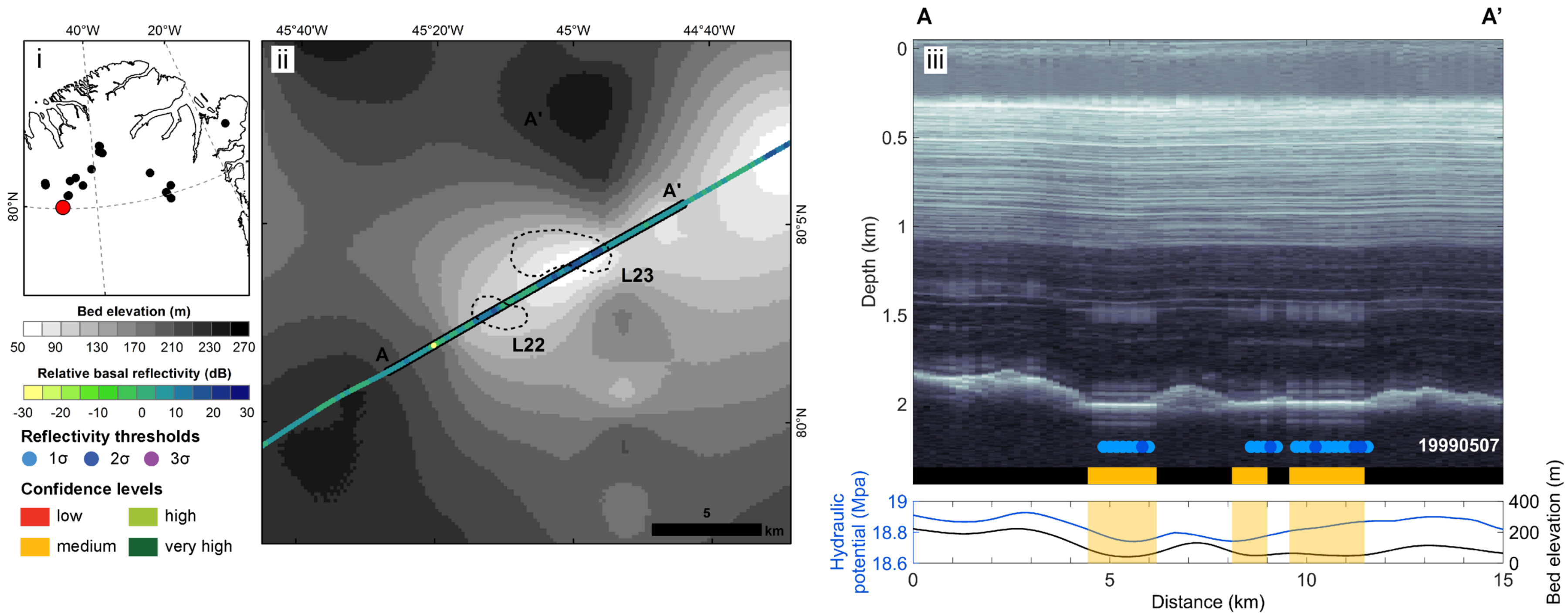

Supplementary Figure 11: Radar evidence for Greenland subglacial lakes. (i) Inset map showing location of subglacial lake L22 and L23 (red) and neighbouring lakes found in this study (black). (ii) Bed topography of the region with relative basal reflectivity along Operation IceBridge flight paths. Estimated lake extent is shown by the dashed line. Radar profile along transect (iii) A-A’ (19990507_01_004). Subglacial lakes are depicted by a bar colour-coded according to the confidence level. Relative basal reflectivity thresholds, based on the statistics of the bed returned power within 20 km of the identified lake (1-3 σ from the mean), are indicated by the blue-purple circles. Lower graphs show bedrock elevation (black) and hydraulic potential (blue).

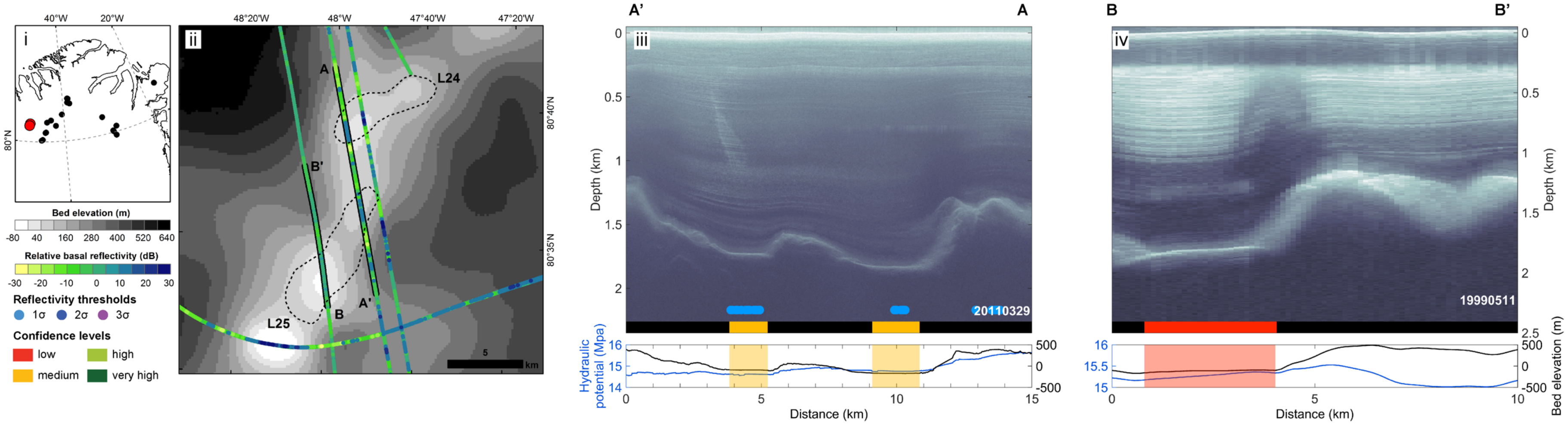

Supplementary Figure 12: Radar evidence for Greenland subglacial lakes. (i) Inset map showing location of subglacial lake L24 and L25 (red) and neighbouring lakes found in this study (black). (ii) Bed topography of the region with relative basal reflectivity along Operation IceBridge flight paths. Estimated lake extent is shown by the dashed line. Radar profile along transect (iii) A-A' (20110329_01_019) and (iv) B-B' (19990511_01_005). Subglacial lakes are depicted by a bar colour-coded according to the confidence level. Relative basal reflectivity thresholds, based on the statistics of the bed returned power within 20 km of the identified lake (1-3σ from the mean), are indicated by the blue-purple circles. Lower graphs show bedrock elevation (black) and hydraulic potential (blue).

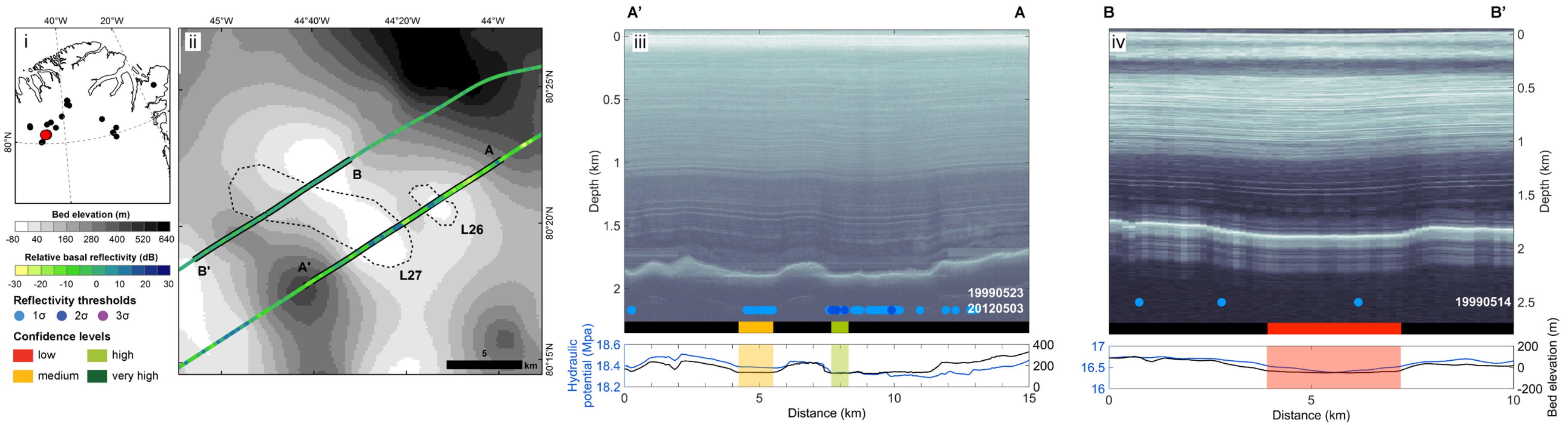

Supplementary Figure 13: Radar evidence for Greenland subglacial lakes. (i) Inset map showing location of subglacial lake L26 and L27 (red) and neighbouring lakes found in this study (black). (ii) Bed topography of the region with relative basal reflectivity along Operation IceBridge flight paths. Estimated lake extent is shown by the dashed line. Radar profile along transect (iii) A-A' (19990523_01_012/ 20120503_03_053) and (iv) B-B' (19990511_01_005). Subglacial lakes are depicted by a bar colour-coded according to the confidence level. Relative basal reflectivity thresholds, based on the statistics of the bed returned power within 20 km of the identified lake (1-3 σ from the mean), are indicated by the blue-purple circles. Lower graphs show bedrock elevation (black) and hydraulic potential (blue).

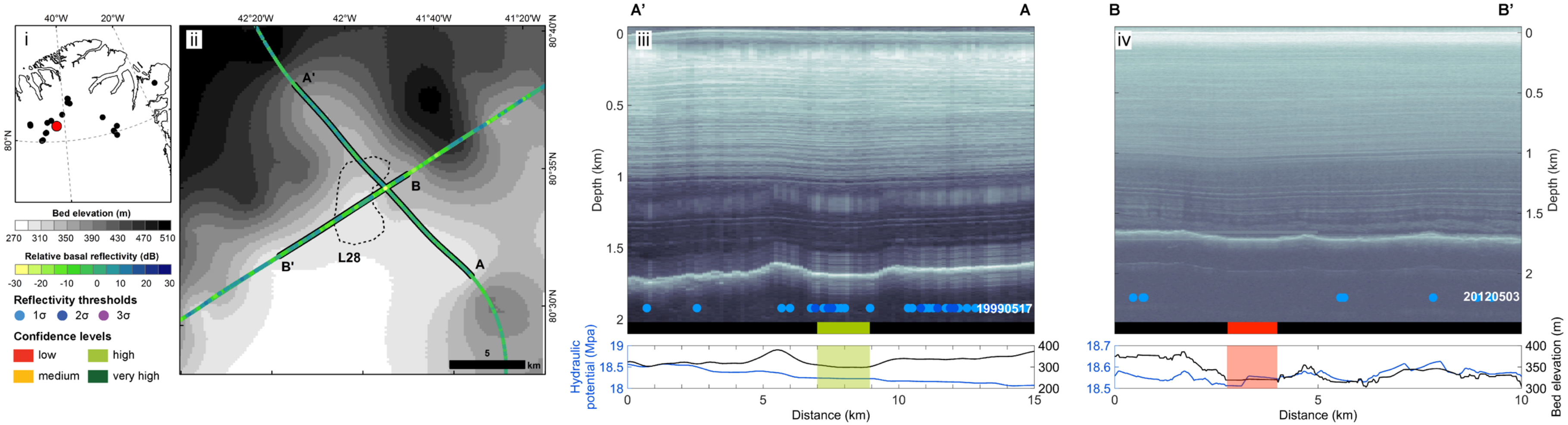

Supplementary Figure 14: Radar evidence for Greenland subglacial lakes. (i) Inset map showing location of subglacial lake L28 (red) and neighbouring lakes found in this study (black). (ii) Bed topography of the region with relative basal reflectivity along Operation IceBridge flight paths. Estimated lake extent is shown by the dashed line. Radar profile along transect (iii) A-A' (19990517_01_001) and (iv) B-B' (20120503_02_052). Subglacial lakes are depicted by a bar colour-coded according to the confidence level. Relative basal reflectivity thresholds, based on the statistics of the bed returned power within 20 km of the identified lake (1-3 σ from the mean),are indicated by the blue-purple circles. Lower graphs show bedrock elevation (black) and hydraulic potential (blue).

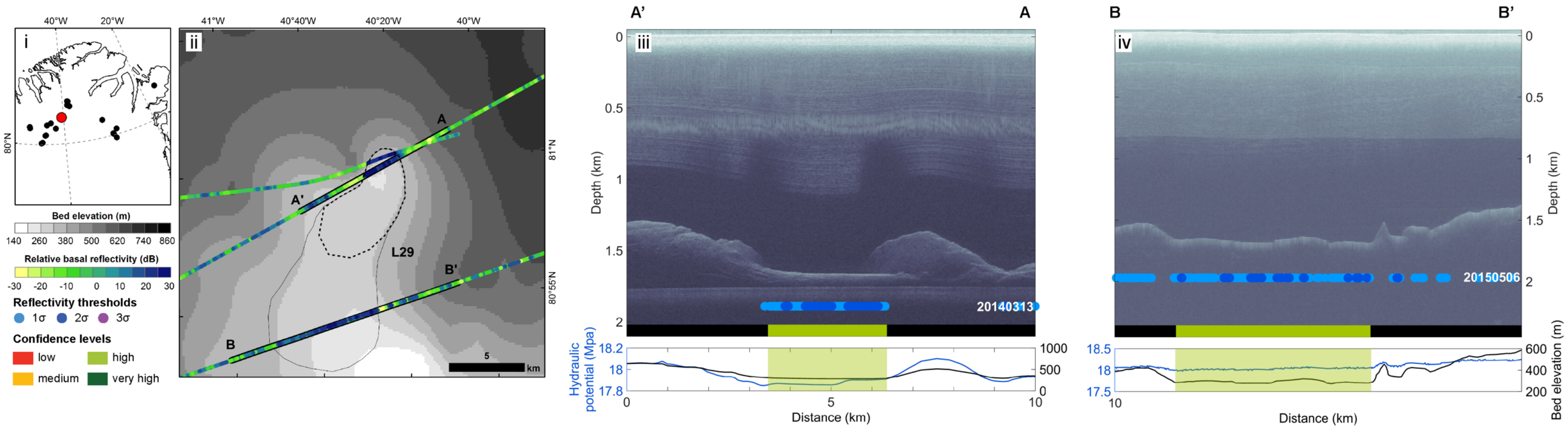

Supplementary Figure 15: Radar evidence for Greenland subglacial lakes. (i) Inset map showing location of subglacial lake L29 (red) and neighbouring lakes found in this study (black). (ii) Bed topography of the region with relative basal reflectivity along Operation IceBridge flight paths. Estimated lake extent is shown by the dashed line. Radar profile along transect (iii) A-A' (20140313_08_001) and (iv) B-B' (20150506_02_026) shows possible further extent. Subglacial lakes are depicted by a bar colour-coded according to the confidence level. Relative basal reflectivity thresholds, based on the statistics of the bed returned power within 20 km of the identified lake (1-3 σ from the mean), are indicated by the blue-purple circles. Lower graphs show bedrock elevation (black) and hydraulic potential (blue).

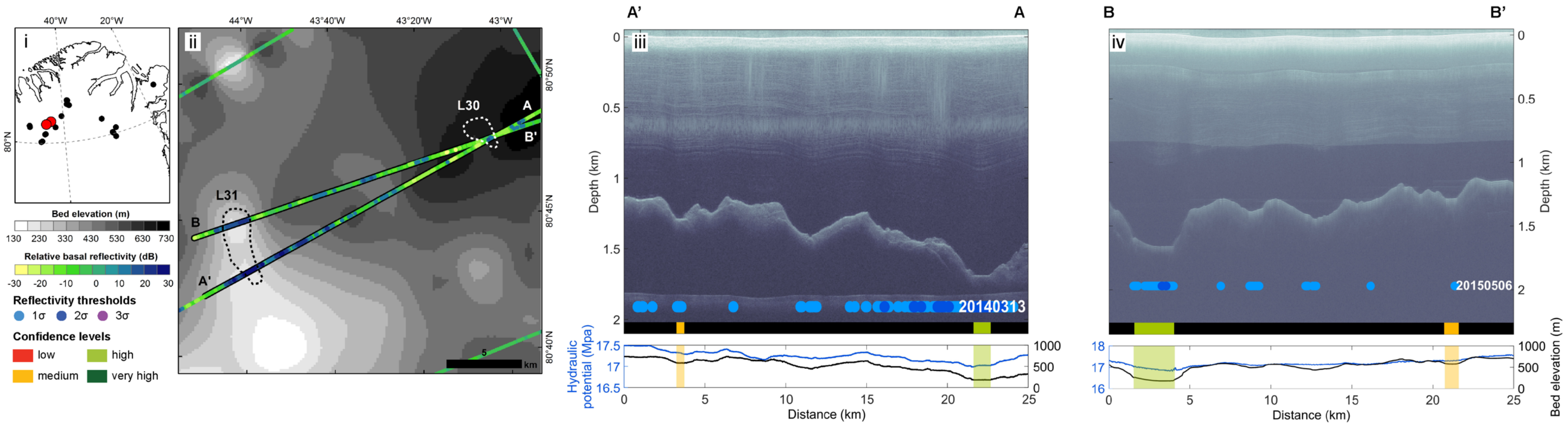

Supplementary Figure 16: Radar evidence for Greenland subglacial lakes. (i) Inset map showing location of subglacial lake L30 and L31 (red) and neighbouring lakes found in this study (black). (ii) Bed topography of the region with relative basal reflectivity along Operation IceBridge flight paths. Estimated lake extent is shown by the dashed line. Radar profile along transect (iii) A-A' (20140313_08_002) and (iv) B-B' (20120506_02_025). Subglacial lakes are depicted by a bar colour-coded according to the confidence level. Relative basal reflectivity thresholds, based on the statistics of the bed returned power within 20 km of the identified lake (1-3 σ from the mean), are indicated by the blue-purple circles. Lower graphs show bedrock elevation (black) and hydraulic potential (blue).

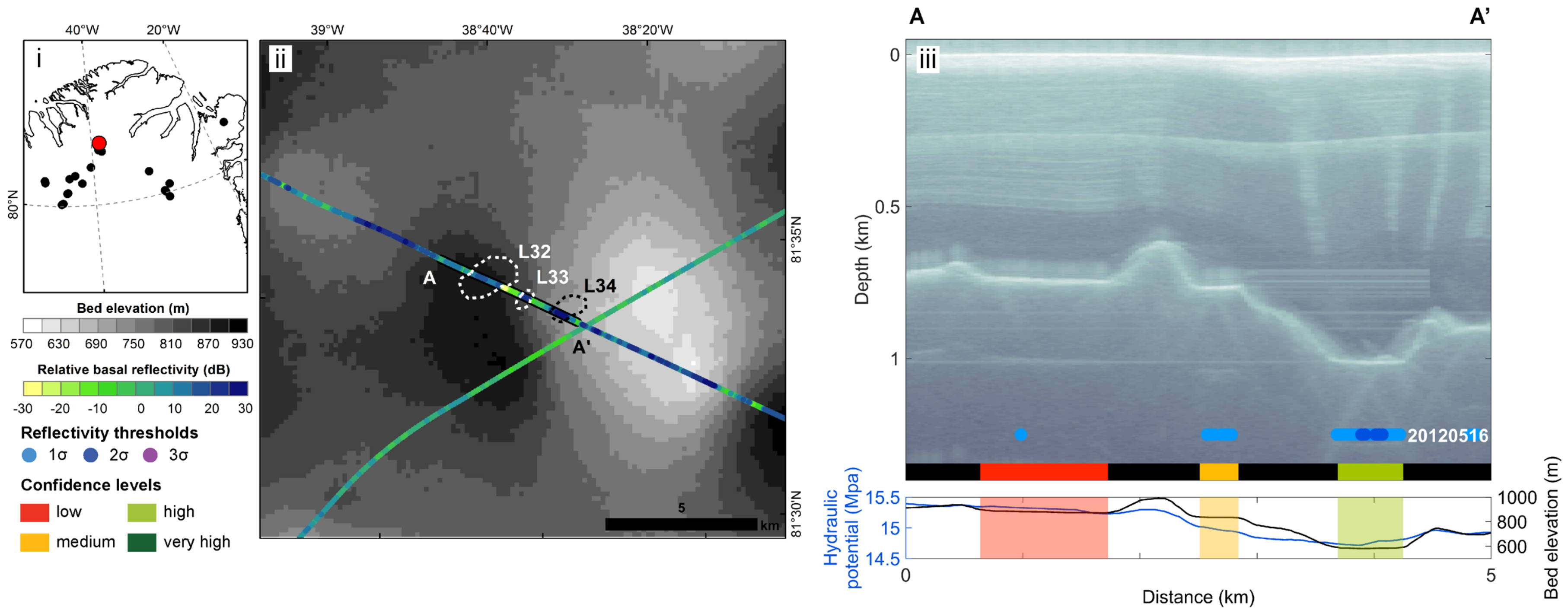

Supplementary Figure 17: Radar evidence for Greenland subglacial lakes. (i) Inset map showing location of subglacial lake L32, L33 and L34 (red) and neighbouring lakes found in this study (black). (ii) Bed topography of the region with relative basal reflectivity along Operation IceBridge flight paths. Estimated lake extent is shown by the dashed line. Radar profile along transect (iii) A-A' (20120516_01_059). Subglacial lakes are depicted by a bar colour-coded according to the confidence level. Relative basal reflectivity thresholds, based on the statistics of the bed returned power within 20 km of the identified lake (1-3 σ from the mean), are indicated by the blue-purple circles. Lower graphs show bedrock elevation (black) and hydraulic potential (blue).

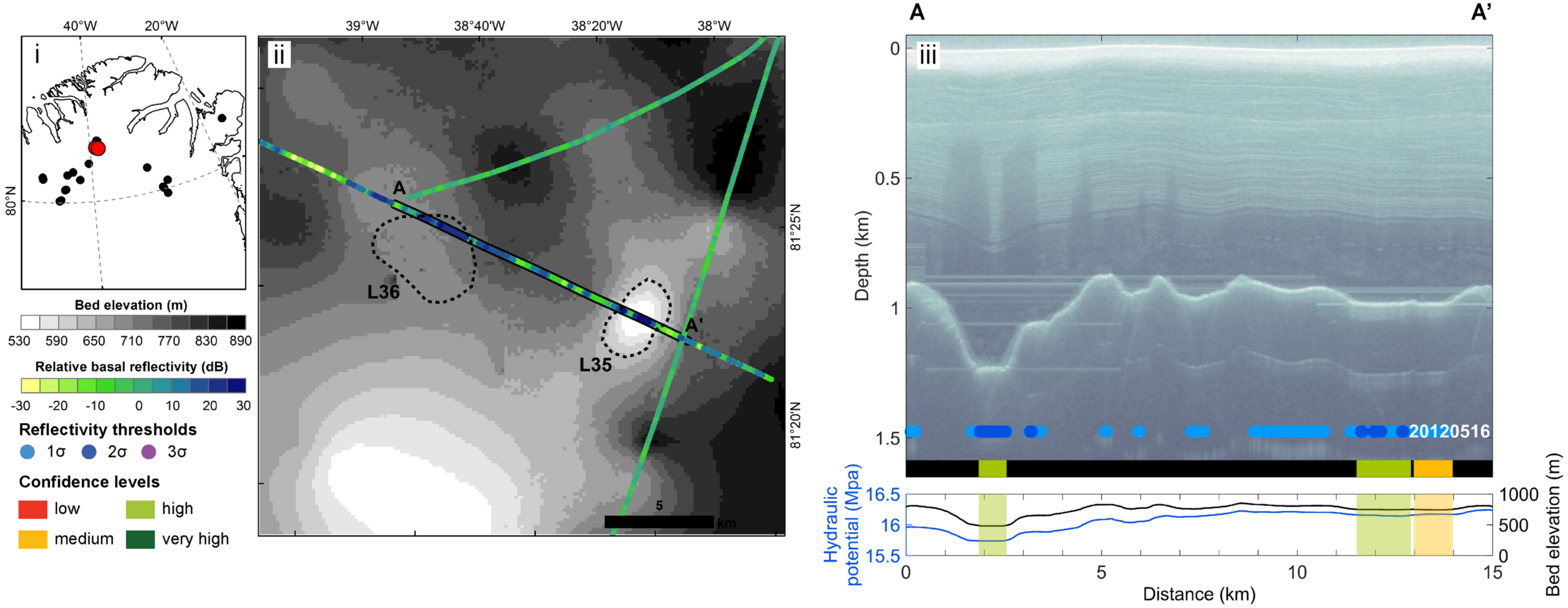

Supplementary Figure 18: Radar evidence for Greenland subglacial lakes. (i) Inset map showing location of subglacial lake L35 and L36 (red) and neighbouring lakes found in this study (black). (ii) Bed topography of the region with relative basal reflectivity along Operation IceBridge flight paths. Estimated lake extent is shown by the dashed line. Radar profile along transect (iii) A-A' (20120516_01_068). Subglacial lakes are depicted by a bar colour-coded according to the confidence level. Relative basal reflectivity thresholds, based on the statistics of the bed returned power within 20 km of the identified lake (1-3 σ from the mean), are indicated by the blue-purple circles. Lower graphs show bedrock elevation (black) and hydraulic potential (blue).

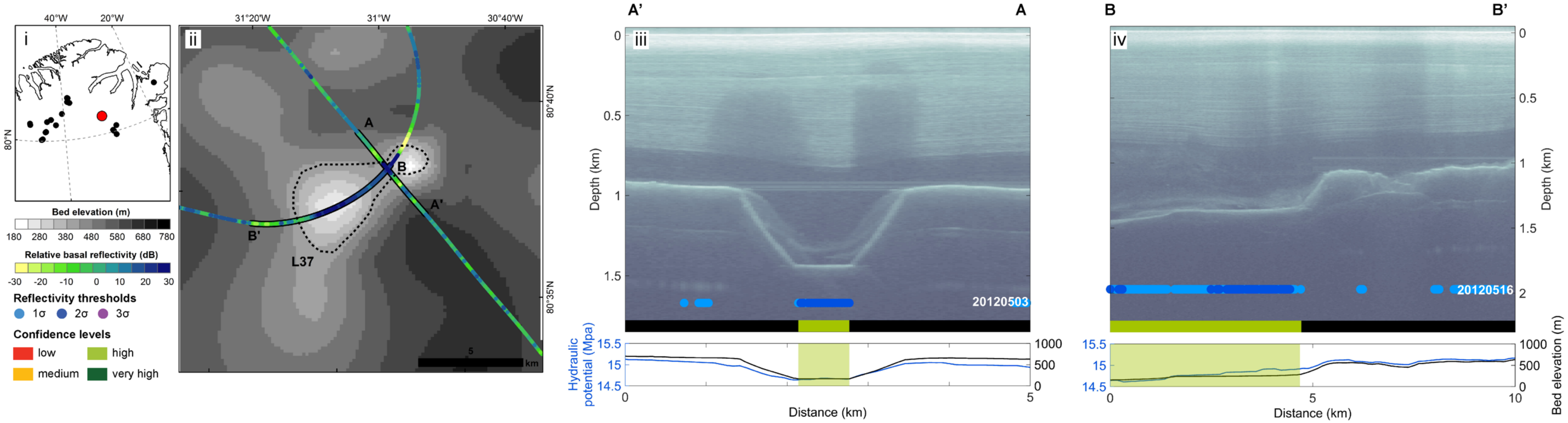

Supplementary Figure 19: Radar evidence for Greenland subglacial lakes. (i) Inset map showing location of subglacial lake L37 (red) and neighbouring lakes found in this study (black). (ii) Bed topography of the region with relative basal reflectivity along Operation IceBridge flight paths. Estimated lake extent is shown by the dashed line. Radar profile along transect (iii) A-A' (20120503_03_037) and (iv) B-B' (20120515_01_064). Subglacial lakes are depicted by a bar colour-coded according to the confidence level. Relative basal reflectivity thresholds, based on the statistics of the bed returned power within 20 km of the identified lake 1-3 σ from the mean), are indicated by the blue-purple circles. Lower graphs show bedrock elevation (black) and hydraulic potential (blue).

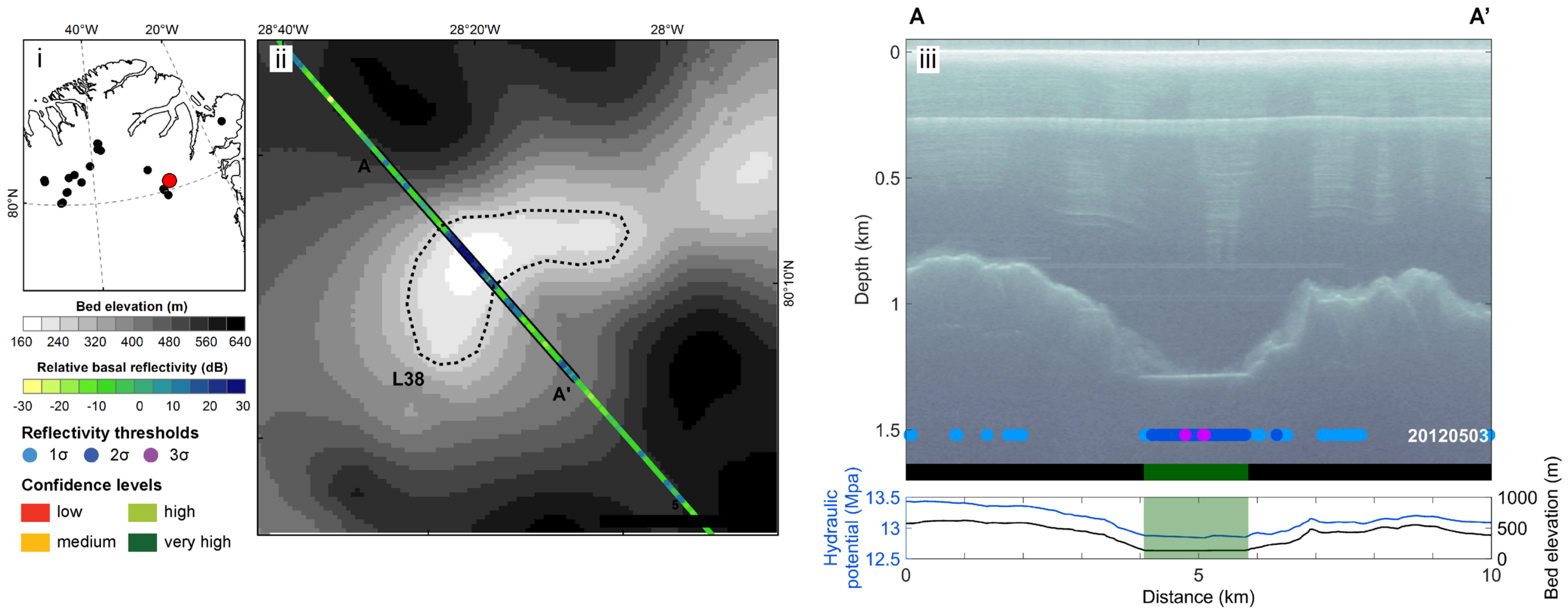

Supplementary Figure 20: Radar evidence for Greenland subglacial lakes. (i) Inset map showing location of subglacial lake L38 (red) and neighbouring lakes found in this study (black). (ii) Bed topography of the region with relative basal reflectivity along Operation IceBridge flight paths. Estimated lake extent is shown by the dashed line. Radar profile along transect (iii) A-A' (20120503_03_030). Subglacial lakes are depicted by a bar colour-coded according to the confidence level. Relative basal reflectivity thresholds, based on the statistics of the bed returned power within 20 km of the identified lake (1-3 σ from the mean), are indicated by the blue-purple circles. Lower graphs show bedrock elevation (black) and hydraulic potential (blue).

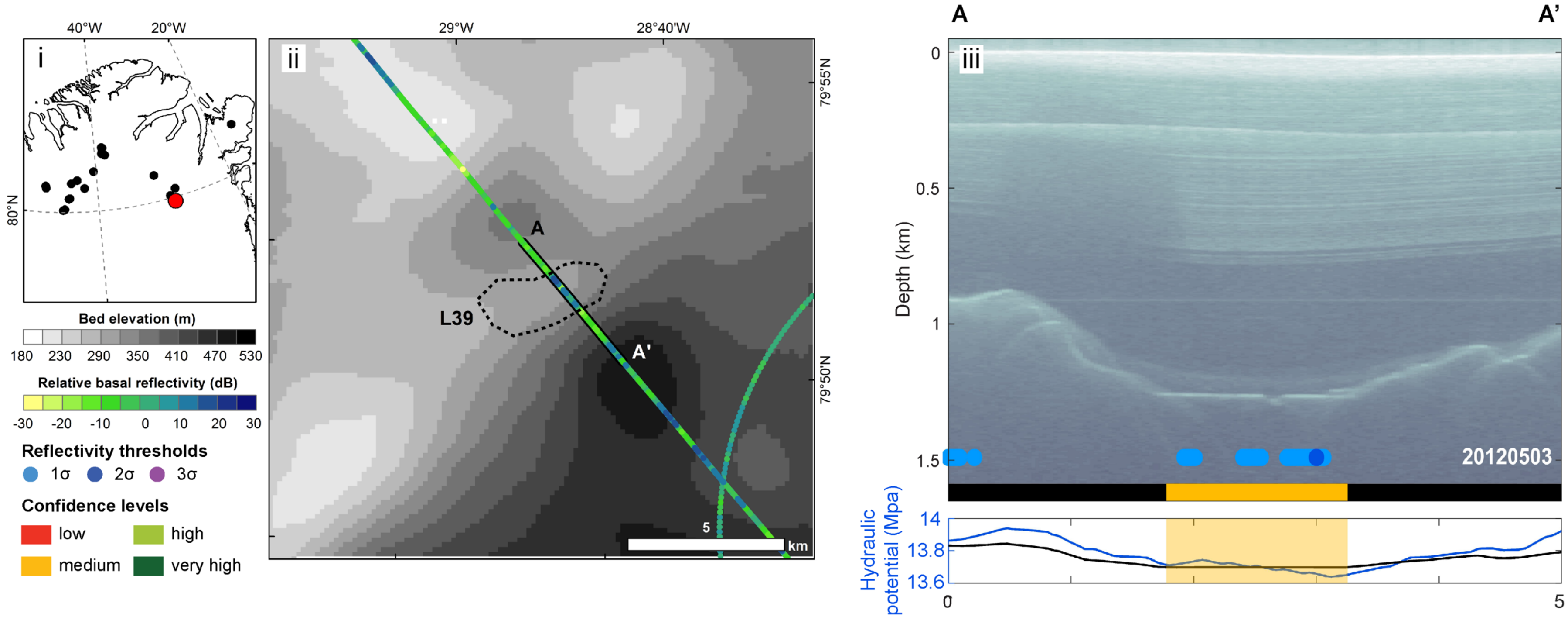

Supplementary Figure 21: Radar evidence for Greenland subglacial lakes. (i) Inset map showing location of subglacial lake L39 (red) and neighbouring lakes found in this study (black). (ii) Bed topography of the region with relative basal reflectivity along Operation IceBridge flight paths. Estimated lake extent is shown by the dashed line. Radar profile along transect (iii) A-A' (20120503_03_034). Subglacial lakes are depicted by a bar colour-coded according to the confidence level. Relative basal reflectivity thresholds, based on the statistics of the bed returned power within 20 km of the identified lake (1-3 σ from the mean), are indicated by the blue-purple circles. Lower graphs show bedrock elevation (black) and hydraulic potential (blue).

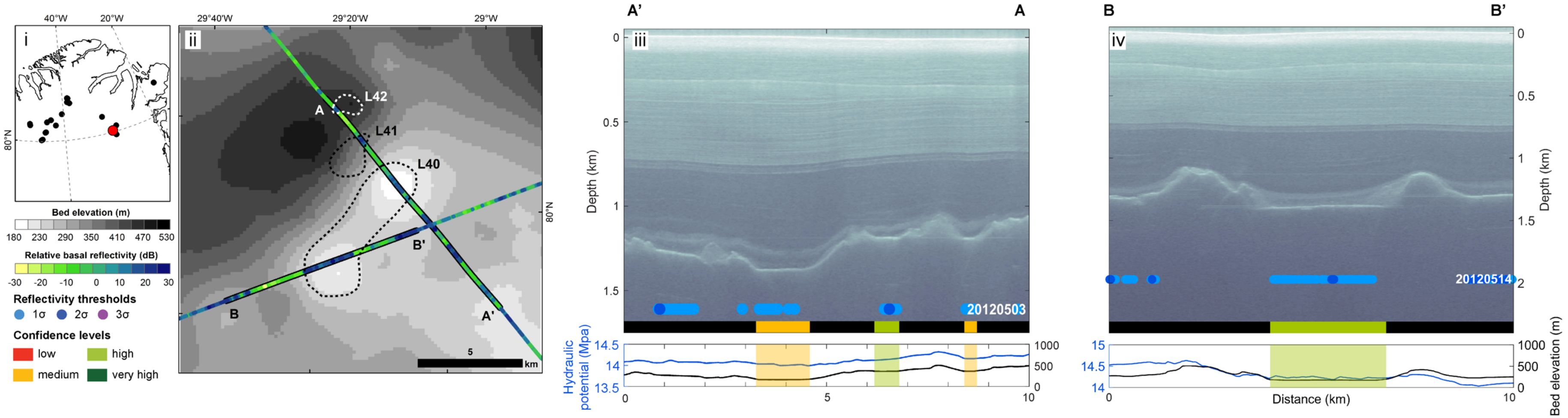

Supplementary Figure 22: Radar evidence for Greenland subglacial lakes. (i) Inset map showing location of subglacial lake L40, L41 and L42 (red) and neighbouring lakes found in this study (black). (ii) Bed topography of the region with relative basal reflectivity along Operation IceBridge flight paths. Estimated lake extent is shown by the dashed line. Radar profile along transect (iii) A-A' (20120503_03_035) and (iv) B-B' (20120514_02_003). Subglacial lakes are depicted by a bar colour-coded according to the confidence level. Relative basal reflectivity thresholds, based on the statistics of the bed returned power within 20 km of the identified lake 1-3 σ from the mean), are indicated by the blue-purple circles. Lower graphs show bedrock elevation (black) and hydraulic potential (blue).

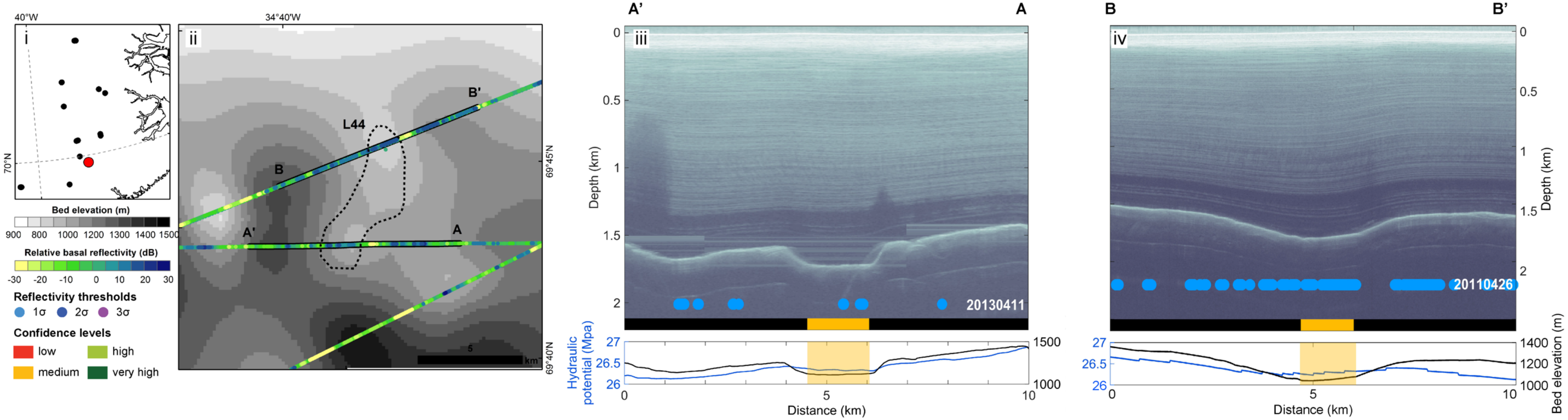

Supplementary Figure 24: Radar evidence for Greenland subglacial lakes. (i) Inset map showing location of subglacial lake L44 (red) and neighbouring lakes found in this study (black). (ii) Bed topography of the region with relative basal reflectivity along Operation IceBridge flight paths. Estimated lake extent is shown by the dashed line. Radar profile along transect (iii) A-A' (20130411_01_050) and (iv) B-B' (20140421_01_015). Subglacial lakes are depicted by a bar colour-coded according to the confidence level. Relative basal reflectivity thresholds, based on the statistics of the bed returned power within 20 km of the identified lake (1-3 σ from the mean), are indicated by the blue-purple circles. Lower graphs show bedrock elevation (black) and hydraulic potential (blue).

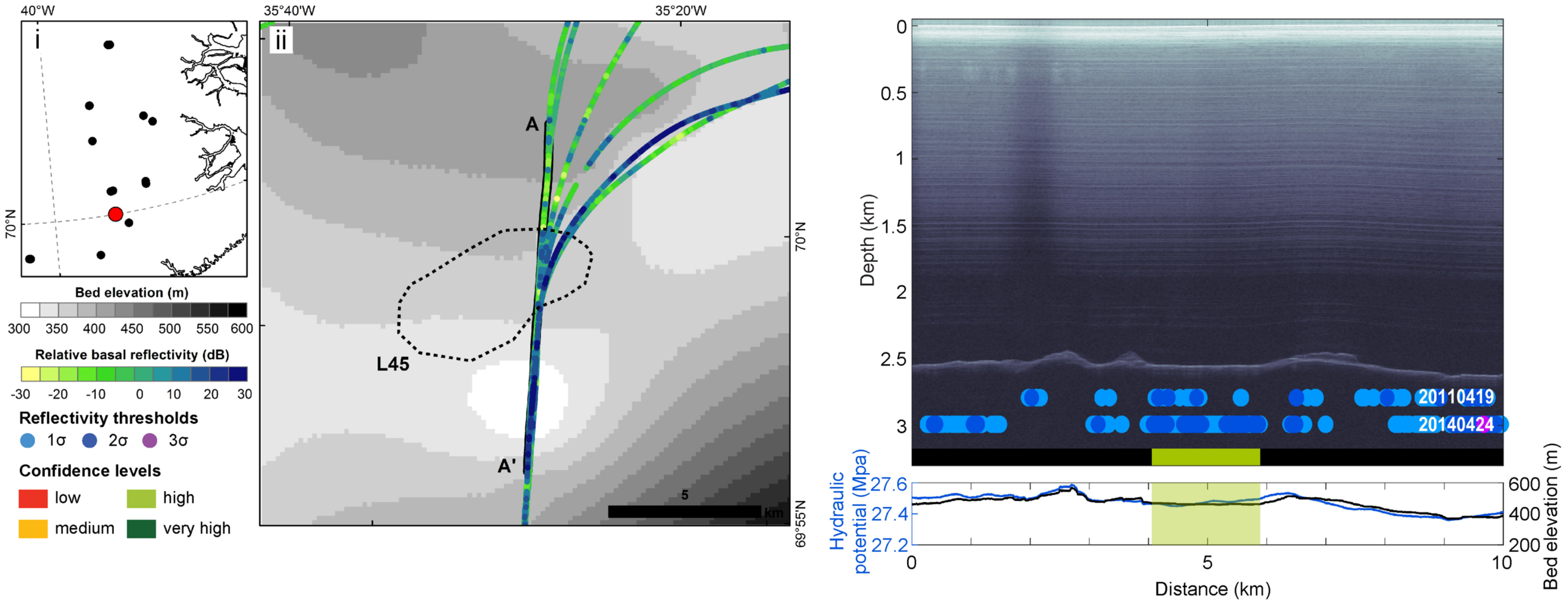

Supplementary Figure 25: Radar evidence for Greenland subglacial lakes. (i) Inset map showing location of subglacial lake L45 (red) and neighbouring lakes found in this study (black). (ii) Bed topography of the region with relative basal reflectivity along Operation IceBridge flight paths. Estimated lake extent is shown by the dashed line. Radar profile along transect (iii) A-A' (20110419_03_002/ 20140424_01_033). Subglacial lakes are depicted by a bar colour-coded according to the confidence level. Relative basal reflectivity thresholds, based on the statistics of the bed returned power within 20 km of the identified lake (1-3 σ from the mean), are indicated by the blue-purple circles. Lower graphs show bedrock elevation (black) and hydraulic potential (blue).

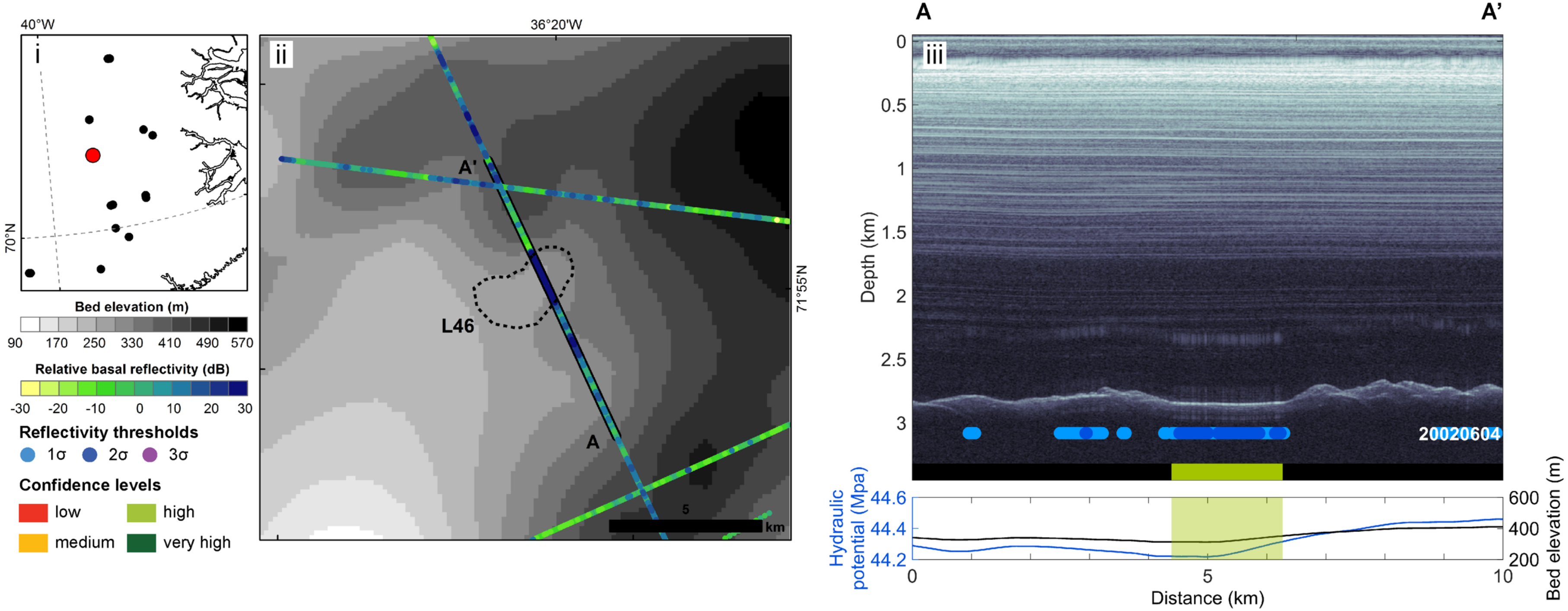

Supplementary Figure 26: Radar evidence for Greenland subglacial lakes. (i) Inset map showing location of subglacial lake L46 (red) and neighbouring lakes found in this study (black). (ii) Bed topography of the region with relative basal reflectivity along Operation IceBridge flight paths. Estimated lake extent is shown by the dashed line. Radar profile along transect (iii) A-A' (20020604_10_007). Subglacial lakes are depicted by a bar colour-coded according to the confidence level. Relative basal reflectivity thresholds, based on the statistics of the bed returned power within 20 km of the identified lake (1-3 σ from the mean), are indicated by the blue-purple circles. Lower graphs show bedrock elevation (black) and hydraulic potential (blue).

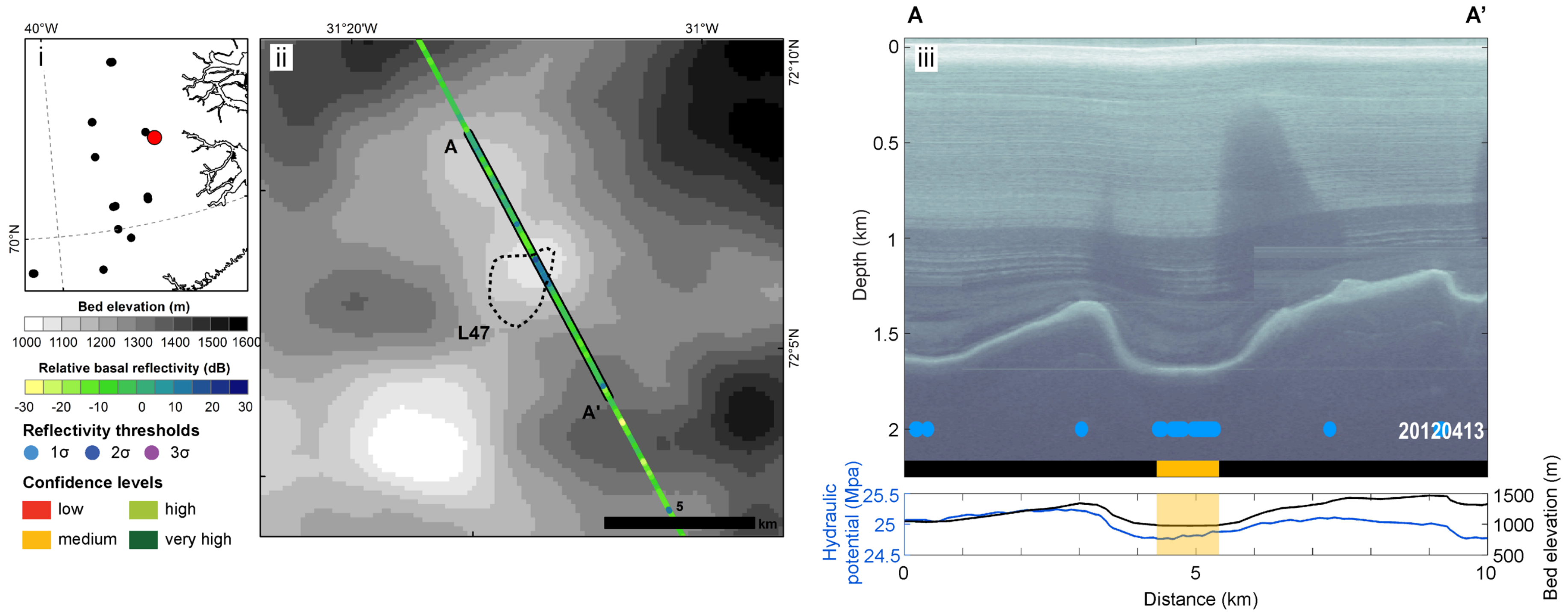

Supplementary Figure 27: Radar evidence for Greenland subglacial lakes. (i) Inset map showing location of subglacial lake L47 (red) and neighbouring lakes found in this study (black). (ii) Bed topography of the region with relative basal reflectivity along Operation IceBridge flight paths. Estimated lake extent is shown by the dashed line. Radar profile along transect (iii) A-A' (20120413_02_036). Subglacial lakes are depicted by a bar colour-coded according to the confidence level. Relative basal reflectivity thresholds, based on the statistics of the bed returned power within 20 km of the identified lake (1-3 σ from the mean), are indicated by the blue-purple circles. Lower graphs show bedrock elevation (black) and hydraulic potential (blue).

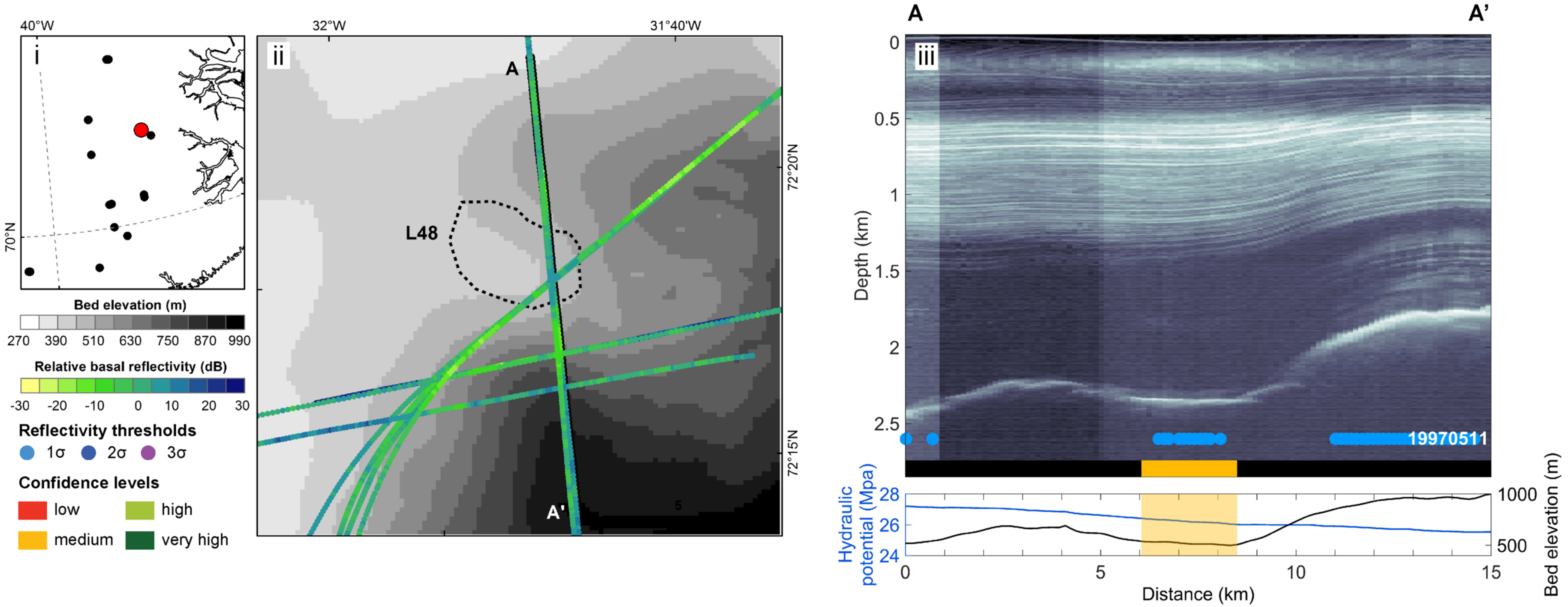

Supplementary Figure 28: Radar evidence for Greenland subglacial lakes. (i) Inset map showing location of subglacial lake L48 (red) and neighbouring lakes found in this study (black). (ii) Bed topography of the region with relative basal reflectivity along Operation IceBridge flight paths. Estimated lake extent is shown by the dashed line. Radar profile along transect (iii) A-A' (19970511_01_005). Subglacial lakes are depicted by a bar colour-coded according to the confidence level. Relative basal reflectivity thresholds, based on the statistics of the bed returned power within 20 km of the identified lake (1-3 σ from the mean), are indicated by the blue-purple circles. Lower graphs show bedrock elevation (black) and hydraulic potential (blue).

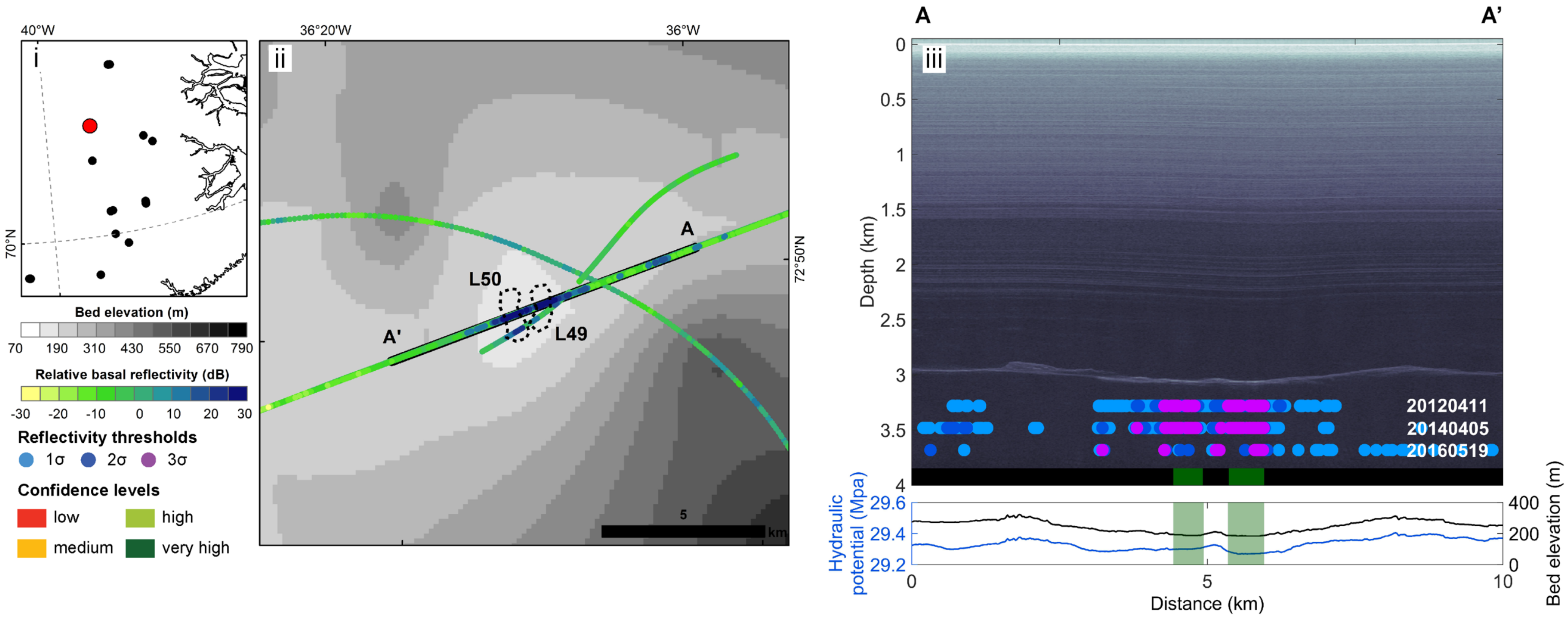

Supplementary Figure 29: Radar evidence for Greenland subglacial lakes. (i) Inset map showing location of subglacial lake L49 and L50 (red) and neighbouring lakes found in this study (black). (ii) Bed topography of the region with relative basal reflectivity along Operation IceBridge flight paths. Estimated lake extent is shown by the dashed line. Radar profile along transect (iii) A-A' (20120411_02_046/ 20140405_01_049/ 20160519_04_005). Subglacial lakes are depicted by a bar colour-coded according to the confidence level. Relative basal reflectivity thresholds, based on the statistics of the bed returned power within 20 km of the identified lake (1-3 σ from the mean), are indicated by the blue-purple circles. Lower graphs show bedrock elevation (black) and hydraulic potential (blue).

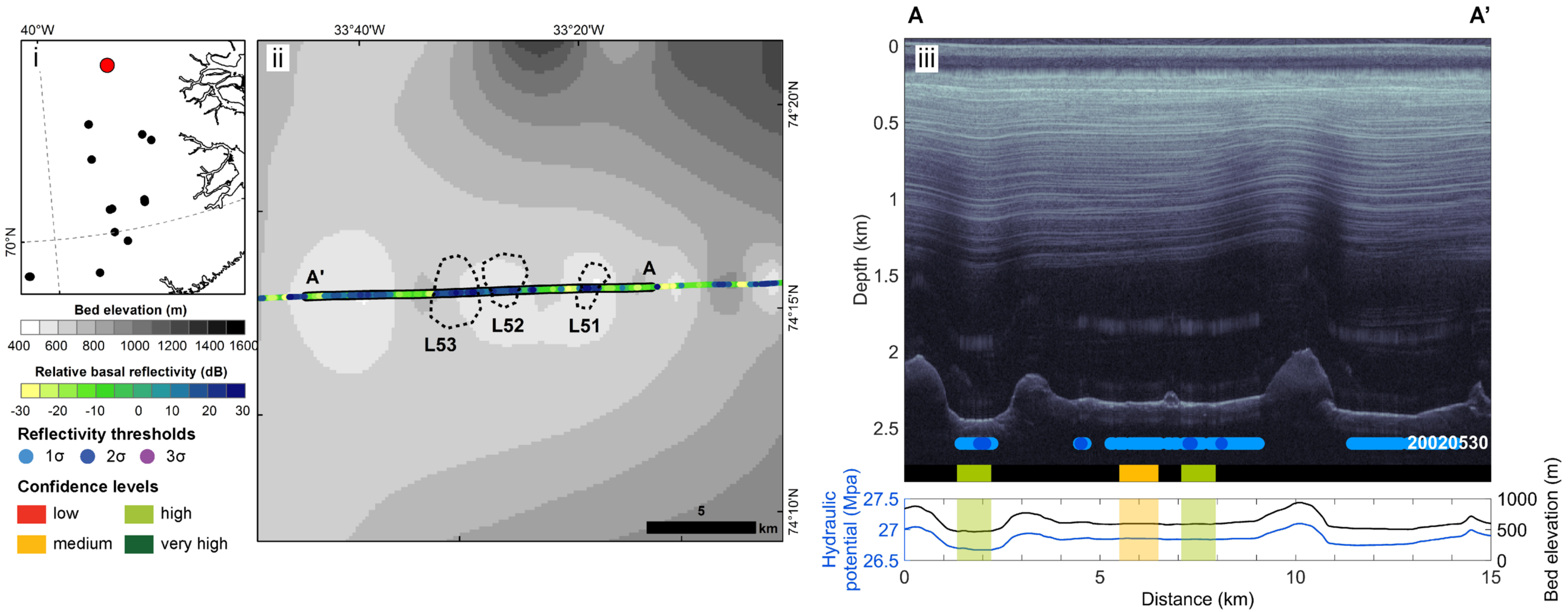

Supplementary Figure 30: Radar evidence for Greenland subglacial lakes. (i) Inset map showing location of subglacial lake L51, L52 and L52 (red) and neighbouring lakes found in this study (black). (ii) Bed topography of the region with relative basal reflectivity along Operation IceBridge flight paths. Estimated lake extent is shown by the dashed line. Radar profile along transect (iii) A-A' (20020530_05_001). Subglacial lakes are depicted by a bar colour-coded according to the confidence level. Relative basal reflectivity thresholds, based on the statistics of the bed returned power within 20 km of the identified lake (1-3 σ from the mean), are indicated by the blue-purple circles. Lower graphs show bedrock elevation (black)and hydraulic potential (blue).

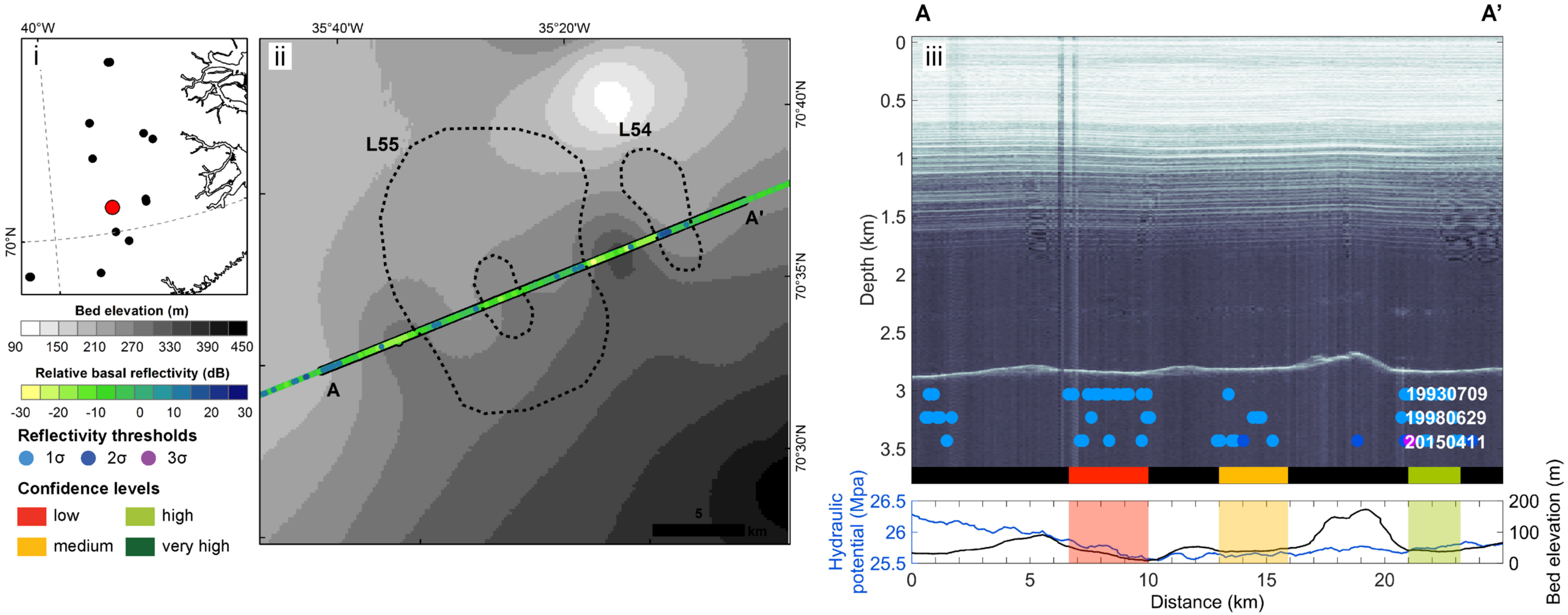

Supplementary Figure 31: Radar evidence for Greenland subglacial lakes. (i) Inset map showing location of subglacial lake L54 and L55 (red) and neighbouring lakes found in this study (black). (ii) Bed topography of the region with relative basal reflectivity along Operation IceBridge flight paths. Estimated lake extent is shown by the dashed line. Radar profile along transect (iii) A-A' (19930709_01_007/ 19980629_01_011/ 20150411_01_016). Subglacial lakes are depicted by a bar colour-coded according to the confidence level. Relative basal reflectivity thresholds, based on the statistics of the bed returned power within 20 km of the identified lake (1-3 σ from the mean), are indicated by the blue-purple circles. Lower graphs show bedrock elevation (black) and hydraulic potential (blue).

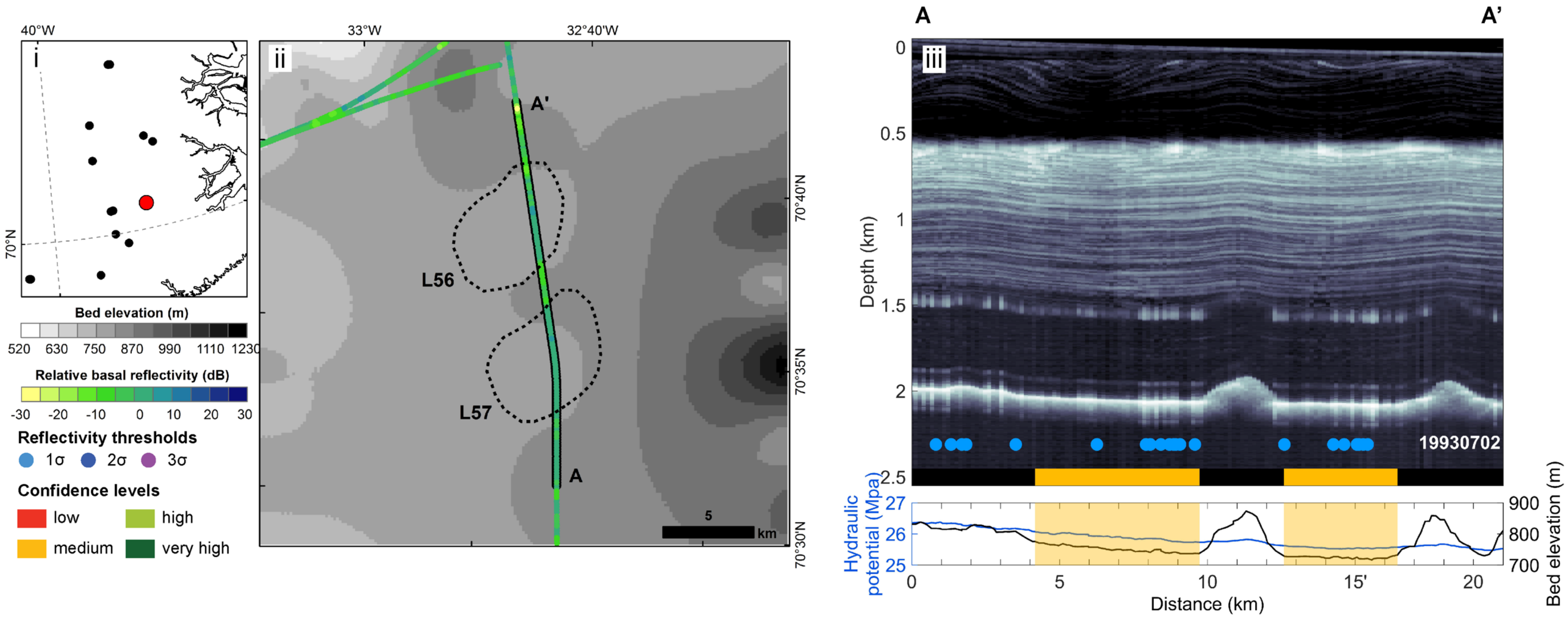

Supplementary Figure 32: Radar evidence for Greenland subglacial lakes. (i) Inset map showing location of subglacial lake L56 and L57 (red) and neighbouring lakes found in this study (black). (ii) Bed topography of the region with relative basal reflectivity along Operation IceBridge flight paths. Estimated lake extent is shown by the dashed line. Radar profile along transect (iii) A-A' (19930702_01_012). Subglacial lakes are depicted by a bar colour-coded according to the confidence level. Relative basal reflectivity thresholds, based on the statistics of the bed returned power within 20 km of the identified lake (1-3 σ from the mean), are indicated by the blue-purple circles. Lower graphs show bedrock elevation (black) and hydraulic potential (blue).

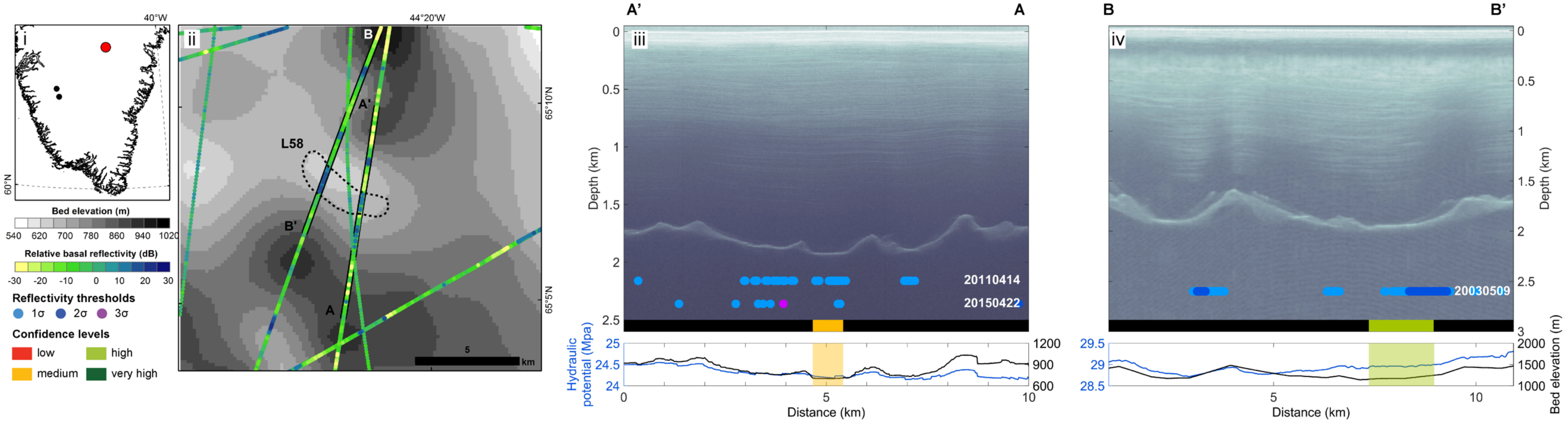

Supplementary Figure 33: Radar evidence for Greenland subglacial lakes. (i) Inset map showing location of subglacial lake L58 (red) and neighbouring lakes found in this study (black). (ii) Bed topography of the region with relative basal reflectivity along Operation IceBridge flight paths. Estimated lake extent is shown by the dashed line. Radar profile along transect (iii) A-A' (20110414_06_002/ 20150422_06_011) and (iv) B-B' (20030509_01_004). Subglacial lakes are depicted by a bar colour-coded according to the confidence level. Relative basal reflectivity thresholds, based on the statistics of the bed returned power within 20 km of the identified lake (1-3 σ from the mean), are indicated by the blue-purple circles. Lower graphs show bedrock elevation (black) and hydraulic potential (blue).

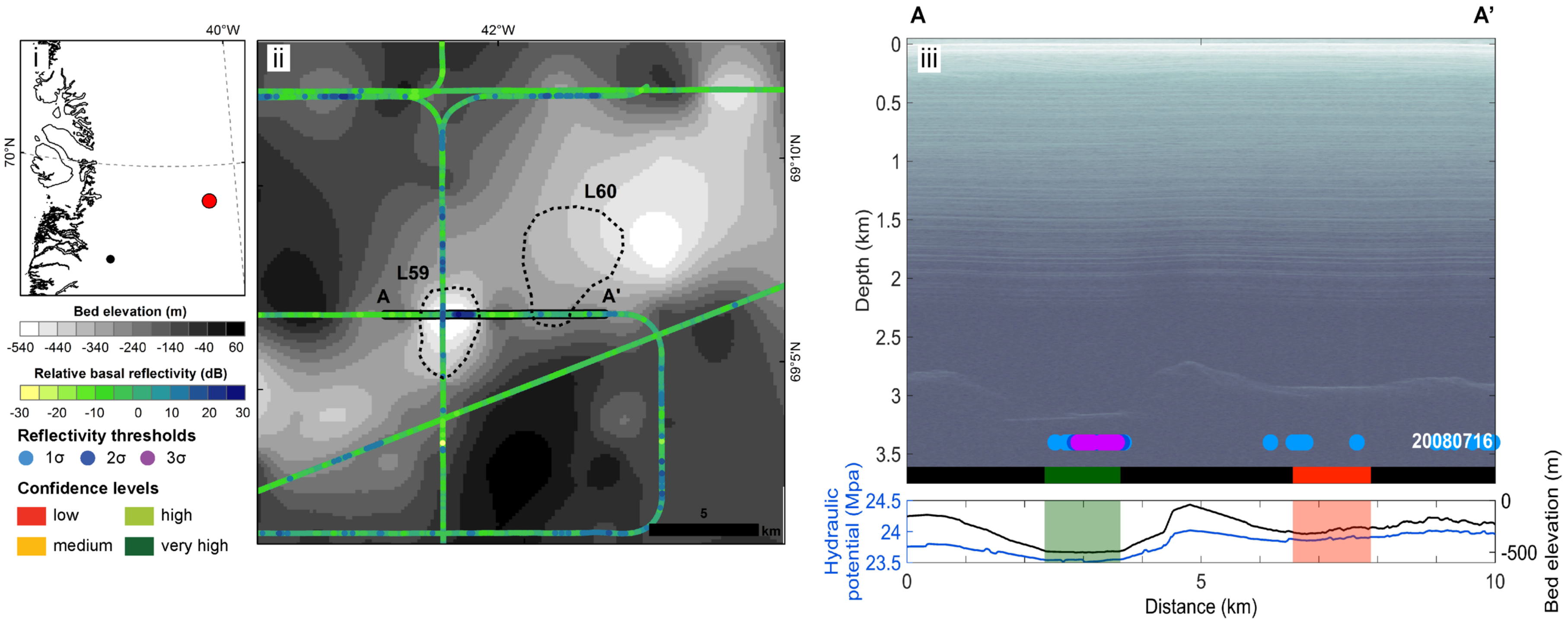

Supplementary Figure 34: Radar evidence for Greenland subglacial lakes. (i) Inset map showing location of subglacial lake L59 and L60 (red) and neighbouring lakes found in this study (black). (ii) Bed topography of the region with relative basal reflectivity along Operation IceBridge flight paths. Estimated lake extent is shown by the dashed line. Radar profile along transect (iii) A-A' (20080716_02_007). Subglacial lakes are depicted by a bar colour-coded according to the confidence level. Relative basal reflectivity thresholds, based on the statistics of the bed returned power within 20 km of the identified lake (1-3 σ from the mean), are indicated by the blue-purple circles. Lower graphs show bedrock elevation (black) and hydraulic potential (blue).

Supplementary Table 1: Inventory of active and stable subglacial lakes beneath the Greenland Ice Sheet, including those in existing literature. This table provides the latitude, longitude, flightpath ID, temporal coverage of IceBridge L2 ATM[1] and ArcticDEM[2] data, estimated minimum persistence based on presence of lake in multiple radar transects and surface elevation change analysis, minimum length of the lake reflector in RES data, thickness of overlying ice[3], geodesic distance from ice divide[4], geodesic distance from the margin, geodesic distance from the ELA[5], mean velocity[6], mean geothermal heat flux[7], predicted basal thermal state[8], bed roughness[9], whether the lake is hydraulically flat, relative basal reflectivity thresholds 1-3 standard deviations from the mean, confidence level ranking per lake.

(Excel file)

Supplementary References

[1]Studinger, M. IceBridge ATM L2 Icessn Elevation, Slope, and Roughness, Version 2. [ILATM2.002], NASA National Snow and Ice Data Center Distributed Active Archive Center, http://dx.doi.org/10.5067/CPRXXK3F39RV (2017).

[2]Porter, C. et al. ArcticDEM, Harvard Dataverse, v2.0, https://doi.org/10.7910/DVN/OHHUKH (2018).

[3]Morlighem, M. et al. BedMachine v3: Complete bed topography and ocean bathymetry mapping of Greenland from multibeam echo sounding combined with mass conservation. Geophys. Res. Lett. 44, 11,051-11,061 (2017).

[4]Rignot, E. & Mouginot, J. Ice flow in Greenland for the International Polar Year 2008-2009. Geophys. Res. Lett. 39, 1–7 (2012).

[5]Fettweis, X. et al. Estimating the Greenland ice sheet surface mass balance contribution to future sea level rise using the regional atmospheric climate model MAR. Cryosphere 7, 469–489 (2013).

[6]Joughin, I., Smith, B., Howat, I. & Scambos, T. MEaSUREs Greenland Ice Sheet Velocity Map from InSAR Data, Version 2, [NSIDC-0478.002], National Snow and Ice Data Center Distributed Active Archive Center., http://dx.doi.org/10.5067/IAGYM8Q26QRE (2017).

[7]Martos, Y. M. et al. Geothermal heat flux reveals the Iceland hotspot track underneath Greenland. Geophys. Res. Lett. 45, 8214–8222 (2018).

[8]MacGregor, J. A. et al. A synthesis of the basal thermal state of the Greenland Ice Sheet. J. Geophys. Res. Earth Surf. 121, 1328–1350 (2016).

[9]Rippin, D. M. Bed roughness beneath the Greenland ice sheet. J. Glaciol. 59, 724–732 (2013).
