# Supplementary material for: Distribution and dynamics of Greenland subglacial lakes
Source: Nat Commun. 2019 Jun 26;10:2810. doi: 10.1038/s41467-019-10821-w (PMC6594964; doi:10.1038/s41467-019-10821-w)
Supplement: Supplementary file 2 — Description of Additional Supplementary Files [file 41467_2019_10821_MOESM2_ESM.pdf]

Description of Additional Supplementary Files

File Name: Supplementary Data 1

Description: Inventory of active and stable subglacial lakes beneath the Greenland Ice Sheet, including those in existing literature. This table provides the latitude, longitude, flightpath ID, temporal coverage of IceBridge L2 ATM and ArcticDEM data, estimated minimum persistence based on presence of lake in multiple radar transects and surface elevation change analysis, minimum length of the lake reflector in RES data, thickness of overlying ice, geodesic distance from ice divide, geodesic distance from the margin, geodesic distance from the ELA, mean velocity, mean geothermal heat flux, predicted basal thermal state, bed roughness, whether the lake is hydraulically flat, relative basal reflectivity thresholds 1-3 standard deviations from the mean, confidence level ranking per lake.
